# Biotic and abiotic factors predicting the global distribution and population density of an invasive large mammal

**DOI:** 10.1038/srep44152

**Published:** 2017-03-09

**Authors:** Jesse S. Lewis, Matthew L. Farnsworth, Chris L. Burdett, David M. Theobald, Miranda Gray, Ryan S. Miller

**Affiliations:** 1 Conservation Science Partners, 5 Old Town Sq, Suite 205, Fort Collins, Colorado, 80524, USA; 2 Colorado State University, Department of Biology, Fort Collins, Colorado, 80524, USA; 3 Conservation Science Partners, 11050 Pioneer Trail, Suite 202, Truckee, California, 96161, USA; 4 United States Department of Agriculture, Animal and Plant Health Inspection Service, Veterinary Services, Center for Epidemiology and Animal Health, Fort Collins, Colorado, 80524, USA

## Abstract

Biotic and abiotic factors are increasingly acknowledged to synergistically shape broad-scale species distributions. However, the relative importance of biotic and abiotic factors in predicting species distributions is unclear. In particular, biotic factors, such as predation and vegetation, including those resulting from anthropogenic land-use change, are underrepresented in species distribution modeling, but could improve model predictions. Using generalized linear models and model selection techniques, we used 129 estimates of population density of wild pigs (Sus scrofa) from 5 continents to evaluate the relative importance, magnitude, and direction of biotic and abiotic factors in predicting population density of an invasive large mammal with a global distribution. Incorporating diverse biotic factors, including agriculture, vegetation cover, and large carnivore richness, into species distribution modeling substantially improved model fit and predictions. Abiotic factors, including precipitation and potential evapotranspiration, were also important predictors. The predictive map of population density revealed wide-ranging potential for an invasive large mammal to expand its distribution globally. This information can be used to proactively create conservation/management plans to control future invasions. Our study demonstrates that the ongoing paradigm shift, which recognizes that both biotic and abiotic factors shape species distributions across broad scales, can be advanced by incorporating diverse biotic factors.

Predicting and mapping species distributions, including geographic range and variability in abundance, is fundamental to the conservation and management of biodiversity and landscapes^1^. The ecological niche defines species-habitat relationships^2^,^3^,^4^ and provides a useful framework for understanding the range and abundance of species in relation to biotic and abiotic factors. Further, niche relationships across local scales can provide novel information about the ecology, conservation, and management of species at macro scales^5^. Most studies evaluating a species’ niche across their distribution focus on presence-absence occurrence data to predict the geographic range^6^; however, conservation and management plans for species can be improved by understanding patterns of population abundance and density across a species’ range^7^. In particular, evaluating population density, compared to occurrence, can reveal novel patterns of species distributions in relation to landscape factors^8^.

There is an ongoing paradigm shift in understanding how biotic and abiotic factors shape species distributions. Until recently, it was widely accepted that abiotic factors, such as temperature and precipitation, played the primary role in shaping distributions of species and biodiversity at broad scales (e.g., regional, continental, global extents) and that biotic factors were most important at fine scales (e.g., site, local extents)^9^,^10^,^11^. It is increasingly recognized, however, that biotic factors are important determinants of species distributions at broad spatial scales, especially when considering biotic interactions^12^,^13^,^14^,^15^,^16^. Although interspecific competition can be an important biotic determinant in species distribution models at broad scales, other forms of biotic interactions, such as predation and symbioses, can also be important determinants^15^,^17^, but have received less attention^18^. In addition, although researchers have evaluated the effects of biotic interactions on geographic range limits^18^, relatively few studies have evaluated how biotic factors influence population density across a species’ range^19^,^20^, which can be more informative in understanding macro-ecological patterns^7^,^21^.

In addition to species interactions, biotic factors related to vegetation can influence species distributions and abundance at broad scales. In particular, anthropogenic land-use change is rarely considered when evaluating species distributions at broad scales; however, given the human footprint globally^22^ and projections for expanding human impacts on the environment^23^,^24^, biotic factors created by human activities are potentially important predictors that can contribute to a better understanding of species distributions^8^. For example, agricultural crops are a dominant biotic factor across continents that are facilitated by human engineering and the redistribution of ecological resources and energy, which can have profound impacts on plant and animal populations across broad extents; agriculture can increase populations for some species through increased food, resource availability, and landscape heterogeneity, or decrease populations due to loss of habitat^25^,^26^,^27^. Ultimately, further evaluation is necessary to understand the relative importance of abiotic and biotic factors in shaping species distributions across broad spatial scales^13^,^15^.

Invasive species are a primary driver of widespread and severe negative impacts to ecosystems, agriculture, and humans across local to global scales^28^. These introduced plants and animals often exhibit broad geographic distributions, can be relatively well studied across local scales, and provide novel opportunities to evaluate broad-scale patterns of niche relationships^29^. Predictions of potential geographic distribution of invasive species can provide critical information that can inform the prevention, eradication, and control of populations, which has been evaluated for many taxa, including plants^30^, amphibians^31^, and invertebrates^32^. However, few studies have predicted the potential ranges and abundance of non-native mammals^33^. Especially for wide-ranging species that can occur across broad extents of landscapes, predictions of how population density varies spatially can provide important information for prioritizing conservation and management actions.

Few species exhibit a global distribution that extends across Europe, Asia, Africa, North and South America, Australia, and oceanic Islands. Besides naturalized animals, such as the house mouse (*Mus musculus*) and brown rat (*Rattus norvegicus*), wild pigs (*Sus scrofa*; other common names include wild boar, wild/feral swine, wild/feral hog, and feral pig) have one of the widest geographic distributions of any mammal; further, it exhibits the widest geographic range of any large mammal^34^, with the exception of humans. The expansive global distribution of wild pigs is attributed to its broad native range in Eurasia and northern Africa, widespread introduction by humans outside its native range, and superior adaptability, where it occurs in a wide variety of ecological communities, ranging from deserts to temperate and tropical environments^35^,^36^, with a corresponding diverse omnivorous diet^37^. Across its non-native range (Fig. 1; Supplementary Methods S1), including North and South America, Australia, sub-Saharan Africa, and many islands, wild pigs are considered one of the 100 most harmful invasive species in the world^38^ due to wide-ranging ecosystem disturbance, agricultural damage, pathogen and disease vectors to wildlife, livestock and people, and social impacts to people and property^39^,^40^,^41^. Wild pigs are therefore a model species to evaluate biotic and abiotic factors associated with population density because they exhibit a global distribution across six continents, are widely studied across much of their native and non-native (i.e., invasive or introduced) ranges, and previous research has indicated that their population density was related to abiotic factors across a continental scale, although it was ambiguous how biotic factors shape their abundance, warranting further study^42^.

To address these ecological questions and understand the relative importance of biotic and abiotic factors in shaping the global distribution of a highly invasive mammal, we evaluated estimates of population density of wild pigs across diverse environments on five continents. Specifically, we (1) evaluate how biotic (i.e., vegetation and predation) and abiotic (i.e., climate) factors (Table 1) shape population density across a global scale and (2) create a predictive distribution map of potential population density across the world. We also compare population density between island and mainland populations. Our results contribute novel insight into the relative roles of biotic and abiotic factors in shaping the distribution of species’ population densities across continental and global scales, particularly relating to human-mediated land-use change, which can provide critical information to management and conservation strategies.

## Results

**Figure 1:**
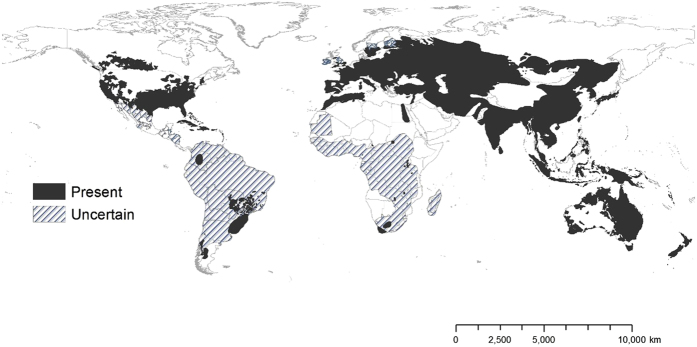
Geographic range of wild pigs across their native and non-native global distribution. Areas of white indicate locations in which wild pigs are likely not present. This map was created using ArcGIS 10.3.1^98^. See Supplementary Methods S1 for a description of methods and citations used for creating the map of wild pig global distribution across its native and non-native ranges.

We compiled 147 estimates of wild pig density (# animals/km^2^), which resulted in 129 estimates of density across their global distribution used in our analyses (Fig. 1; Supplementary Table S2). Some areas contained > 1 density estimate, and these were averaged. Population density of wild pigs was higher on islands (n = 11) compared to on the mainland (n = 118) (t = 4.72, df = 10.93, p < 0.001; Supplementary Figure S3). For the untransformed density estimates, mean population density for on the mainland equaled 2.75 (se = 0.38) and islands equaled 18.52 (se = 4.15). The highest estimates of population density occurred on islands, which reached upwards of 40 wild pigs/km^2^ (Supplementary Table S2). Due to differences in population density between islands and on the mainland, we used density estimates from mainland populations in our subsequent analyses.

**Figure 2:**
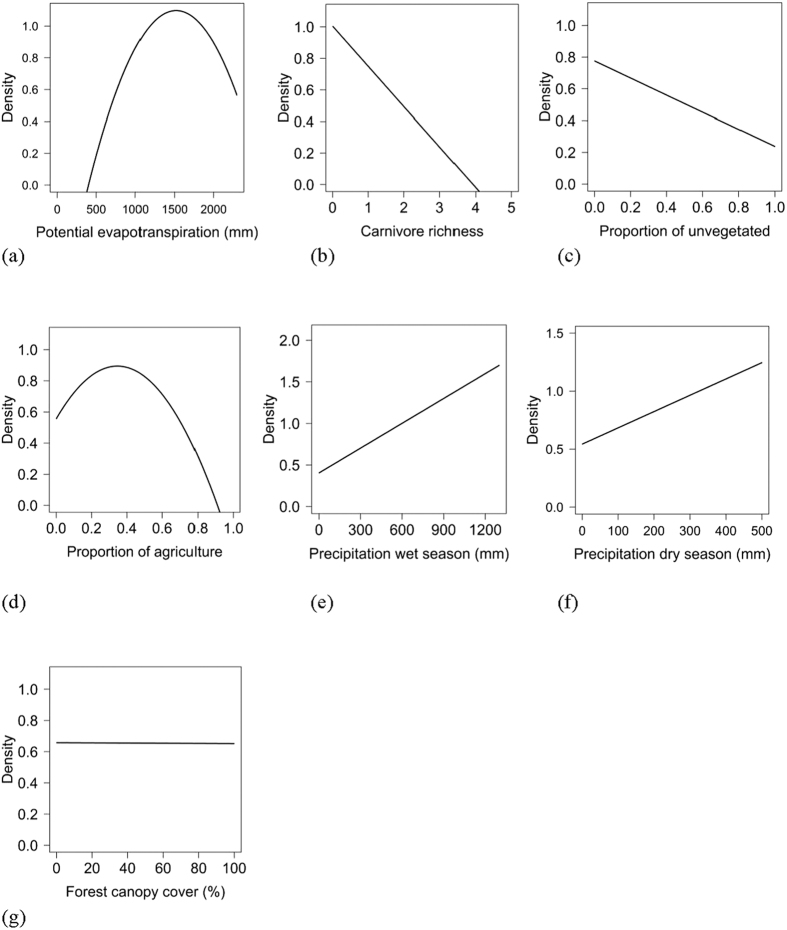
Relationships of biotic and abiotic factors with population density (natural log scale; #/km^2^) of wild pigs, including potential evapotranspiration (**a**), large carnivore richness (**b**), unvegetated (**c**), agriculture (**d**), precipitation during the wettest season (**e**), precipitation during the driest season (**f**), and forest canopy cover (**g**).

Population density was influenced by both biotic and abiotic factors across the global distribution (Tables 2 and 3; Supplementary Table S4). The suite of best models all included combinations of biotic and abiotic factors (Table 2) and the top model (AIC_c_ = 237.94; model weight = 0.68; adjusted R^2^ = 0.55) had > 1,000 times more support as the best approximating model than the top model considering only abiotic factors (AIC_c_ = 311.30; model weight = 7.94 × 10^−17^) (Supplementary Table S4). The variables with the greatest importance included potential evapotranspiration, large carnivore richness, precipitation during the wet and dry seasons, unvegetated area, and agriculture, which also exhibited 95% confidence intervals that did not overlap zero (Table 3). Density was greatest at moderate levels of potential evapotranspiration and agriculture, decreased with large carnivore richness and amount of unvegetated area, and increased with precipitation during the wet and dry seasons (Fig. 2); percent forest cover was unsupported in models when considering the suite of variables in analyses.

**Figure 3:**
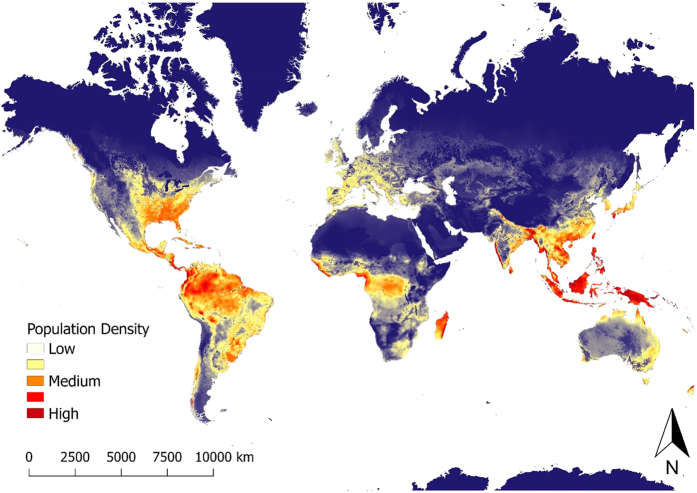
Map of predicted population density of wild pigs for habitat occurring across the world. For terrestrial environments, areas of white represent low density (1 individual/km^2^), orange moderate density (6 individuals/km^2^), and dark red high density (≥11 individuals/km^2^). Maps were created using Google Earth Engine^80^ and QGIS 2.14.3^90^. See Supplementary Figure S5 for finer scale maps of predicted population density of wild pigs for Europe, Asia, Africa, Australia, North America, and South America.

Using the full model-averaged results of parameter estimates, we created a predictive map of global wild pig population density (Fig. 3; Supplementary Figure S5). Wild pig populations were predicted to occur at low to high population densities across all continents, including large areas of land where wild pigs are currently absent. The highest predicted densities occurred in southeastern, eastern, and western North America, throughout Central America, northern, eastern, and southwestern South America, western, southern, and eastern Eurasia, throughout Indonesia, central and southern Africa, and northern and southeastern Australia (Fig. 3; Supplementary Figure S5). Results of k-fold cross validation demonstrated that the model had good predictive ability with a mean squared prediction error (MSPE) of 0.22 and a Pearson’s correlation between observed and predicted values of 0.80 (t = 17.711, df = 181, p-value < 0.001).

## Discussion

Population density of an invasive large mammal was strongly influenced by both biotic and abiotic factors across its global distribution. Consistent with the prediction that abiotic factors drive broad-scale patterns of species distribution, potential evapotranspiration (PET) and precipitation variables were important predictors of population density on a global scale. In addition, contributing to growing evidence that biotic factors are also important determinants of broad-scale patterns of species distributions, both biotic interactions and vegetation played important roles in predicting the distribution of wild pig populations globally. Further, land-use change mediated by human activities strongly predicted the broad-scale distribution of an invasive large mammal. Consistent with previous studies evaluating how population density of ungulates varied across broad scales, both bottom-up (resource-related) and top-down (predation) factors influenced the distribution of wild pig populations^19^,^42^,^43^. Ultimately, wild pig populations across their global distribution appeared to respond to biotic and abiotic factors related to plant productivity, forage and water availability, cover, predation, and anthropogenic land-use change.

Using both biotic and abiotic factors to evaluate broad-scale species distributions can create more realistic maps of range and density with better predictive ability^16^,^44^, which can better inform management and conservation strategies for species. For example, population density of wild pigs was highest in landscapes with moderate levels of agriculture and PET, lower large carnivore richness and amount of unvegetated area, and greater precipitation during the wet and dry seasons. Using these relationships, we created a predictive map of population density across the world, which can be used to manage existing populations and predict areas where wild pig populations are likely to expand or invade if given the opportunity. Ultimately, this information can be used to prioritize management activities in areas at risk of invasion and with expanding populations.

Abiotic factors, such as temperature and precipitation, are consistently found to be primary determinants of species distributions at broad scales^11^. Potential evapotranspiration can be especially informative for understanding broad-scale ecological patterns^45^, such as species distributions. This was supported in our research where PET was the most important predictor of population density across the global distribution of wild pigs. Potential evapotranspiration is highly correlated with temperature variables, thus indicating that wild pig density was greatest at relatively moderate temperatures and density was lower in areas exhibiting extreme low and high temperatures. In addition, the strong support of precipitation variables in our models is consistent with the association of wild pigs with vegetation cover, forage, and water^36^. In particular, precipitation likely facilitates rooting behavior by wild pigs by softening the soil substrate^46^.

Biotic factors were among the most supported variables predicting population density across a global scale. Our results indicated that the presence of large carnivores can influence wild pig population density. Large carnivore richness was strongly supported in our models and exhibited a negative relationship with wild pig density; as the number of large carnivore species increased, wild pig density decreased, which is consistent with studies in Eurasia and Australia^42^,^47^,^48^. In addition, interspecific competition can influence the distribution of species and it has been hypothesized that wild pigs have not extensively invaded wildlands in some regions of sub-Saharan Africa due to the presence of other pig species that exhibit similar niches^49^. Although competition with other species might influence wild pig populations and their distribution^49^,^50^,^51^, in other cases wild pigs are reported to spatially and temporally partition habitat use to reduce niche overlap with potential competitors^52^,^53^,^54^ and not show evidence for interference competition with related mammals (e.g., species within the suborder Suiformes), such as native peccary species^55^, thus, it is unclear how interspecific interactions influence wild pig populations across their global distribution. Further, understanding potential interspecific competition for invasive species can be especially challenging in non-native habitat because invaders have not coevolved with competitors or predators and thus it is difficult to predict which species will be subordinate or dominant in potential competitive interactions or how competition might influence species distributions in unoccupied habitat^17^,^18^,^56^. Because it was unknown how competitive interactions between wild pigs and other species might influence their distribution, particularly outside their native range, competition was not included in our analyses. To understand how competition between non-native and native species influences species distributions, field studies evaluating interspecific competition are necessary across the wild pig’s native and non-native geographic range, particularly across local spatial scales.

Although biotic interactions between animals are the primary biotic factors evaluated in species distribution models at broad scales, the role of plant communities has received less consideration. In particular, anthropogenic land-use change increasingly influences vegetation communities across continents and warrants a better understanding for how human activities are shaping broad-scale distributions of plant and animal populations^22^,^24^. For example, agriculture is a dominating land cover type across continents^23^,^25^, which can potentially benefit species distributions in at least two ways. Agriculture can (1) increase population density within areas of a species’ current geographic range through supplemental food and increased resource availability and (2) allow geographic ranges to expand by creating habitat in areas that were previously unsuitable. In contrast, as agriculture increasingly dominates landscape patterns at broad extents, cover and other resources correspondingly decrease, which can negatively impact the geographic range and population density of some species. Our results demonstrate that agriculture can produce both positive and negative effects on populations, depending on the levels of agriculture. At intermediate levels of agriculture, population density of wild pigs was greatest, likely due to an optimal mix of food and cover. Whereas, at high levels of agriculture, population density decreased precipitously, which was likely a result of inadequate cover. Our results indicate that heterogeneous landscapes with a mix of agriculture and cover will support the greatest populations of wild pigs, which is consistent with broad-scale patterns of wild pig populations in North America and Eurasia^57^,^58^,^59^. Due to relatively high predicted population densities of wild pigs inhabiting heterogeneous landscapes, these regions would likely experience the greatest crop damage, leading to high economic loss to farmers.

Forest is considered a key habitat type preferred by wild pigs^59^,^60^. In univariate analyses, forest was an important positive predictor of wild pig density (β = 0.170, se = 0.056). When considering additional predictor variables in our models, however, forest was relatively unimportant in predicting wild pig density, which is also consistent when evaluating wild pig occurrence over broad scales^57^. Thus, the interpretation of how forest influences the distribution of wild pigs must be considered in the context of other variables included in models, where abiotic factors might adequately explain forest distribution (see discussion below). However, as predicted, vegetation and cover play a strong role in predicting wild pig density; as the amount of unvegetated area increased across the landscape, wild pig population density decreased, which is consistent with geographic distribution maps of wild pigs^61^.

In some systems, abiotic factors can be stronger predictors of species distributions, than biotic factors, because of high correlations between these two factors^62^. Our study indicated that both factors can be important predictors of species distributions, potentially because abiotic factors may poorly predict biotic factors stemming from human activities. In addition, human influences might weaken the correlation between abiotic and biotic factors. For example, humans can significantly reduce the number of large carnivores in an area^63^, although these species would be predicted to occur across broad areas based on abiotic factors and historic biotic conditions. In addition, human land use change can lead to unpredictable biotic patterns in relation to abiotic factors, such as through agricultural landscape conversion. Although soil types might support crop production, many agricultural areas occur in arid landscapes requiring irrigation of water and application of fertilizer to maintain production^25^. Thus, agricultural crops could not grow in many areas based on broad-scale climate factors alone, and therefore, abiotic factors can be poor predictors of agricultural practices in some regions. Indeed, there likely are other examples where abiotic and biotic factors may exhibit low correlation in some systems (e.g., location of human activities and development, altered interspecific interactions due to human activities, and other forms of anthropogenic land use change). Ultimately, it can be useful to consider biotic factors in species distribution models that might be poorly predicted by abiotic factors due to human activities.

Additional biotic factors that can influences species distributions on a broad scale, particularly invasive species, include the role of humans in distributing the founding individuals of new populations. For example, invasive wild pig populations have arisen across several continents recently through human activities. Illegal translocations by humans for hunting purposes can facilitate the long-distance expansion of wild pig populations into new areas^64^,^65^,^66^, which is currently a primary source of new populations globally^39^,^41^. Further, in countries such as Canada, Brazil, and Sweden, wild pig farms were the propagule source for recent populations of wild pigs across broad regions, which are currently spreading into new areas^67^,^68^,^69^. Indeed, propagule pressure (i.e., the number of individuals introduced and release events) determines both the likelihood of invasive species becoming established, as well as the rate of geographic range expansion^60^,^70^. In addition, invasive species that exhibit r-selected characteristics (e.g., early maturity, short generation time, and high fecundity) can be more likely to successfully invade novel landscapes^71^. Even at low population densities, invasive species with high reproductive output are more likely to establish populations in areas of lower quality habitat^72^. Given that wild pigs are one of the most fecund large mammals (e.g., mean litter sizes ranging from 3.0 to 8.4 piglets per sow with the potential for >1 litter annually)^36^, their reproductive characteristics might increase the probability of establishment and enable them to compensate for small population sizes when introduced into novel environments across a range of habitat qualities.

Population density, compared to presence-absence occurrence, can provide more informative conclusions of species distributions in relation to biotic and abiotic factors^7^,^8^. For example, although large carnivores likely do not exclude wild pigs from habitat across broad scales, our study revealed they can influence abundance. However, occurrence of species would remain constant across varying population densities, unless it resulted in species exclusion. Ultimately, population densities can provide more detailed information about species distributions, which can better inform conservation and management plans and policy^7^. Studies analyzing presence-only data with logistic regression and Maximum Entropy (MaxEnt) models have examined methods to address spatial sampling bias^73^,^74^,^75^ and additional evaluations would be useful for studies using population density data with multiple linear regression. Further, global analyses of population genetics could be used to identify groups and the proportion of wild and domestic genes across wild pig populations, which could be used to incorporate population structure into analyses to better understand population characteristics.

Predicting species distributions provides critical information to the management and conservation of biodiversity, especially for controlling invasive species. Without intensive management actions, our study predicts that there is strong potential for wild pigs to expand their geographic range and further invade expansive areas of North America, South America, Africa, and Australia. Although wild pigs currently occupy broad regions of predicted habitat in their non-native range, many regions of predicted habitat are currently unoccupied and may be at high risk for future invasion. These areas might warrant increased surveillance by local, state, and federal agencies to counter the establishment of populations. Although attention in unoccupied areas that are predicted to support high densities of wild pigs might warrant priority for countering population introductions, wild pigs can persist in relatively low quality habitat (e.g., arid and/or cold regions) and these areas also warrant attention to halt invasions. Given the potential for wild pig populations to rapidly expand once established^36^, predictions of potential population density in unoccupied habitat can provide critical information to land managers, which can be used to proactively develop management plans to prevent introductions and control or eradicate populations if they become introduced.

## Methods

### Density Estimates

To evaluate the population density (i.e., number of individuals per unit area) of wild pigs throughout their global distribution, we compiled density estimates from the literature throughout its native and non-native ranges across each continent and island for which data were available (Supplementary Table S1). Previous research evaluated how population density of wild pigs varied across western Eurasia^42^ and we incorporated these 54 estimates of population density into our analysis. In addition, we followed the methodological recommendation of Melis *et al*.^42^. to average data when multiple estimates were available for >1 season or year at a study area. Island populations typically exhibit higher population density compared to mainland populations^76^,^77^. We thus compared estimates of wild pig population density between island and mainland populations; if population density for islands was significantly higher than on the mainland, we focused on only evaluating mainland populations in subsequent analyses.

Models evaluating and predicting species distributions can be improved by including areas of absence (a.k.a., pseudo-absence or background locations) or zero density to sample the full range of available landscape conditions^1^ to predict the potential range of a species, absence locations should occur outside the environmental domain of the species, but within a reasonable distance of the species’ geographic range^78^. Because wild pigs have occurred within their native range for thousands of years, we assumed that populations were at equilibrium and the species had colonized available habitat associated with its geographic distribution. Thus, regions adjacent to its native distribution that were classified as unoccupied were assumed to be unsuitable for population persistence due to unfavorable environmental conditions. In addition, spatial sampling bias (i.e., uneven sampling across geographic extents) can be addressed by increasing the number of background locations in areas with greater sampling^73^,^74^. The majority of density estimates used in our study occurred within the wild pig’s native range of Europe and Asia and we focused sampling of background locations associated with this region. To include locations with estimates of zero density in our analyses, we used a three-step approach. First, we created a buffered region that occurred across the area between 100–1000 km around the boundary of the wild pig’s native range^79^. Next, we calculated the spatial extent of the native range and buffered regions. Lastly, accounting for the area of each region, we selected a random sample of locations within the buffered region that was proportional to the number of estimates used in the native terrestrial range of wild pigs. Based on this approach, we used 65 locations of zero density in our analyses that occurred across central Russia, Mongolia, western China, Saudi Arabia, and northern African countries. Zero density estimates were used in analyses relating wild pig density to landscape variables and excluded when comparing population density between island and mainland populations.

### Landscape Variables

We considered a suite of biotic and abiotic landscape variables, which were divided into vegetation, predation, and climate factors (Table 1) that we hypothesized to influence population density of wild pigs. We used landscape variables that were available globally and, where possible, over long time periods (i.e., estimates averaged over several decades) that coincide with the density estimates we compiled for our analyses. Geospatial data layers were acquired through either Google Earth Engine^80^ or were downloaded from online sources (Table 1).

The biotic factors that we evaluated included agriculture, broadleaf forest, enhanced vegetation index (EVI), forest canopy cover, difference in the proportion between forest and agriculture (to characterize landscape heterogeneity), normalized difference vegetation index (NDVI), large carnivore richness, and unvegetated area (Table 1). We expected a positive relationship between density and all vegetation factors, except unvegetated area, due to their association with increased food availability, plant productivity, and cover. In addition, we expected a quadratic relationship between population density and agriculture because we predicted density to be greatest at moderate levels of agriculture (due to a mix of cover and food) and low at high levels of agriculture (due to a lack of adequate cover). Finally, we expected a negative relationship between population density and large carnivore richness.

The abiotic factors that we evaluated included two measures of ecological energy regimes, actual evapotranspiration (the amount of water loss from evaporation and transpiration, which is related to plant productivity) and potential evapotranspiration (PET; the amount of evaporation and transpiration that would occur with a sufficient water supply, considering solar radiation, air temperature, humidity, and wind speed;^45^). Actual evapotranspiration is a measure of water-energy balance and potential evapotranspiration is considered a measure of ambient energy and often highly correlated with temperature variables^81^. Although evapotranspiration variables can include elements of biotic (i.e., transpiration from plants) and abiotic (i.e., climate and water) factors, they were classified as abiotic for our analyses. In addition, we evaluated precipitation during dry and wet seasons, and annually, and temperature during summer and winter, and annually (Table 1). We predicted a positive relationship between density and precipitation variables due to associated increases in forage, water, and cover and quadratic relationships between density and evapotranspiration and temperature variables due to expected peak densities at intermediate levels and low densities at low and high levels.

### Modeling

We used data from the wild pig’s native and non-native range in our modeling. Although niche shifts between a species’ native and non-native range appear to be uncommon and it is often assumed that species exhibit niche stasis or conservatism^30^,^82^,^83^,^84^ through space and time, models that use data only from a species’ native range can exhibit poor predictive power in the species’ non-native range^85^,^86^,^87^. Therefore, it is important to include data from the species’ entire distribution to increase the predictive ability of models across both the native and non-native ranges^32^,^88^,^89^. Because wild pigs have been established across much of their non-native range for an extended period of time (e.g., typically greater than a century), we assumed that populations used in our analyses had achieved a localized equilibrium with their environment.

All geospatial data layers were evaluated using QGIS^90^ and Google Earth Engine^80^ and statistical analyses were conducted using R^91^. Because there is uncertainty about the exact location of studies and the scale in which processes might influence wild pig densities, we evaluated multiple scales for each covariate using 10, 20, and 40 km radius buffers around the location of each density estimate (Table 1). Thus a moving window approach was conducted so that each pixel within a spatial layer summarized the landscape within the buffered radius. To determine the best scale for analyses we used a multi-criteria approach. First, variables were centered and scaled to improve model fit^92^. Next, we considered quadratic relationships for landscape factors that were predicted to exhibit a curvilinear pattern (Table 1). Last, we selected the best scale and relationship for each covariate based on wild pig ecology, model comparisons using Akaike’s Information Criterion corrected for small sample size AICc;^93^, and plots of residuals. Once the appropriate scale was determined for each variable (Table 1), we evaluated the Pearson correlation among all variables and excluded highly correlated variables (r > 0.70) from our final analysis.

We used multiple linear regression to evaluate how population density was influenced by our final suite of biotic and abiotic factors (Table 1). The distribution of density estimates were right skewed, thus we log-transformed density estimates using the natural logarithm^42^. To compare the relative importance of biotic and abiotic factors and to determine parameter estimates of variables, we ranked all possible models using AIC_c_, model-averaged parameter estimates (i.e., full conditional), and calculated variable importance values^93^,^94^,^95^. We used model weights and evidence ratios to evaluate if biotic factors improved model fit by comparing models including only abiotic factors to models also including biotic factors. Model averaged parameter estimates were used to create a predictive global map of wild pig density (1 km^2^ resolution). This map displays the maximal potential density of wild pigs in relation to the biotic and abiotic factors used in our modeling and reflects predicted densities that would be achieved if wild pigs had access to all landscapes, their movements were unrestricted, and management activities did not suppress populations. We validated our model using mean squared prediction error (MSPE)^96^ and k-fold cross validation and selected the number of bins based on Huberty’s rule of thumb (k = 4)^97^.

## Additional Information

**How to cite this article:** Lewis, J. S. *et al*. Biotic and abiotic factors predicting the global distribution and population density of an invasive large mammal. *Sci. Rep.* **7**, 44152; doi: 10.1038/srep44152 (2017).

**Publisher's note:** Springer Nature remains neutral with regard to jurisdictional claims in published maps and institutional affiliations.

## Supplementary Material

Supplementary Files

## Figures and Tables

**Table 1:** Description of landscape variables considered in analyses evaluating how biotic and abiotic factors influenced wild pig population density across their global distribution.

Landscape Variable	Category, Description of Variable, and Calculation Method	Predicted Relationship	Supporting Citations for Prediction	Data Source
Agriculture	Biotic/Vegetation; all agricultural crop lands; proportional area within 10 km radius buffer	Positive, quadratic	Geisser and Reyer^99^, Honda^59^, Ballari and Barrios-García^37^, Morelle and Lejeune^100^	Global Land Cover by National Mapping Organizations (GLCNMO) 2008; cropland cover types
Enhanced Vegetation Index (EVI)*	Biotic/Vegetation; plant productivity; mean value within 10 km radius buffer	Positive	Plant productivity: Melis, *et al*.^42^.	Google Earth Engine; Landsat 5 TM 32-Day EVI Composite 1984–2012
Forest Canopy Cover	Biotic/Vegetation; all forest over 5 m; mean value of canopy cover within 10 km radius buffer	Positive	Honda^59^, Morelle, *et al*.^60^.	Google Earth Engine; Hansen Global Forest Change v1.0 year 2000
Forest Minus Agriculture*	Biotic/Vegetation; difference between the proportion of forest and agriculture within 10 km radius buffer	Positive	See forest (classified as present or absent for this variable) and agriculture descriptions	See data sources for forest canopy cover and agriculture
Normalized Difference Vegetation Index (NDVI)*	Biotic/Vegetation; plant productivity; mean value in 10 km radius buffer	Positive	Plant productivity: Melis, *et al*.^42^.	Google Earth Engine; Landsat 5 TM 32-Day NDVI Composite 1984–2012
Unvegetated Area	Biotic/Vegetation; cover types lacking vegetation, including bare, snow and ice, and urban; proportion within 10 km radius buffer	Negative	Plant productivity: Melis, *et al*.^42^.	Global Land Cover by National Mapping Organizations (GLCNMO) 2008; sparse vegetation, bare area, urban, and snow and ice cover types
Large Carnivore Richness	Biotic/Predation; number of terrestrial large carnivores presented by Ripple, *et al*.^63^, excluding the panda bear and adding the dingo; mean value within 40 km radius buffer	Negative	Woodall^47^, Jedrzejewska, *et al*.^50^., Sweitzer^101^, Ickes^48^, Melis, *et al*.^42^., Mayer and Brisbin^36^, Massei, *et al*.^58^.	Large carnivore distributions from IUCN^79^, Dingo distribution in Australia^102^
Actual Evapotranspiration*	Abiotic/Climate; combination of evaporation of water and transpiration from plants; mean value within 40 km radius buffer	Positive, quadratic	Fisher, *et al*.^45^.	Global High-Resolution Soil-Water Balance: 1950–2000; Trabucco and Zomer^103^
Potential Evapotranspiration	Abiotic/Climate; combination of evaporation of water and transpiration from plants; mean value within 40 km radius buffer	Positive, quadratic	Fisher, *et al*.^45^.	Global High-Resolution Soil-Water Balance: 1950–2000; Trabucco and Zomer^103^
Precipitation Annual *	Abiotic/Climate; total precipitation during annual period; mean value within 40 km radius buffer	Positive	Woodall^47^, Weltzin, *et al*.^104^ but see Geisser and Reyer^99^	Bioclim WorldClim World Climate Data – Bio 12 Annual Precipitation (mm); 1950–2000
Precipitation Driest Season	Abiotic/Climate; total precipitation during driest 3 month annual period; mean value within 40 km radius buffer	Positive	Mortality related to periods of low precipitation, especially during summer^105^	Bioclim WorldClim World Climate Data – Bio 17 Precipitation of Driest Quarter (mm); 1950–2000
Precipitation Wettest Season	Abiotic/Climate; total precipitation during wettest 3 month annual period; mean value within 40 km radius buffer	Positive	Woodall^47^, Weltzin, *et al*.^104^ but see Geisser and Reyer^99^	Bioclim WorldClim World Climate Data – Bio 16 Precipitation of Wettest Quarter (mm); 1950–2000
Temperature Annual*	Abiotic/Climate; mean temperature over annual period; mean value within 40 km radius buffer	Positive, quadratic	Jedrzejewska, *et al*.^50^.	Bioclim WorldClim World Climate Data – Bio 1 Annual Mean Temperature (C); 1950–2000
Temperature Summer*	Abiotic/Climate; mean temperature over warmest 3 month annual period; mean value within 40 km radius buffer	Positive, quadratic	Geisser and Reyer^99^, McClure, *et al*.^57^., but see Groves^106^	Bioclim WorldClim World Climate Data – Bio 10 Mean Temperature of Warmest Quarter; 1950–2000
Temperature Winter*	Abiotic/Climate; mean temperature over coldest 3 month annual period; mean value within 10 km radius buffer	Positive, quadratic	Bieber and Ruf^107^, Geisser and Reyer^99^, Melis, *et al*.^42^., Honda^59^, McClure, *et al*.^57^., but see Groves^106^	Bioclim WorldClim World Climate Data – Bio 11 Mean Temperature of Coldest Quarter; 1950–2000

An asterisk (*) indicates landscape variables that were excluded from the final analyses due to high correlation with other variables.

**Table 2:** Model selection results using Akaike Information Criteria (AIC_c_) from analyses evaluating how population density of wild pigs was related to biotic and abiotic factors.

Potential Evapotranspiration	Large Carnivore	Precipitation Wet Season	Unvegetated	Agriculture	Precipitation Dry Season	Forest	K	AIC_c_	Δ AIC_c_	weight	log(L)
*	*	*	*	*	*		10	237.94	0.00	0.68	−108.33
*	*	*	*	*	*	*	11	240.18	2.24	0.22	−108.32
*	*	*	*	*			9	243.00	5.06	0.05	−111.98
*	*	*	*	*		*	10	244.40	6.46	0.03	−111.56
*	*	*	*		*		8	246.14	8.20	0.01	−114.65
*	*	*	*		*	*	9	248.20	10.26	0.00	−114.58
*	*	*		*	*		9	248.25	10.31	0.00	−114.60
*	*	*		*	*	*	10	249.14	11.20	0.00	−113.93
*	*	*		*		*	9	252.95	15.01	0.00	−116.95
*	*	*	*				7	253.93	15.99	0.00	−119.64

A “*” in the covariate columns indicates whether the variable was included in the model. K is the number of variables included in the model. Note that Potential Evapotranspiration and Agriculture include both main and quadratic effects (thus accounting for two parameters for each of these variables). Only the top 10 models are reported. See Supplementary Table S4 for AIC_c_ model selection results of all possible variable combinations.

**Table 3:** Model selection results for parameters evaluating how population density of wild pigs is influenced by biotic and abiotic factors.

	Potential Evapotranspiration	Large Carnivore	Precipitation Wet Season	Unvegetated	Agriculture	Precipitation Dry Season	Forest
Variable Importance Values	1.00	1.00	1.00	0.99	0.98	0.92	0.25
Parameter Estimate (Standard Error)	m: 0.443 (0.056) q: −0.226 (0.046)	−0.243 (0.043)	0.233 (0.055)	−0.203 (0.061)	m: 0.236 (0.076)q: −0.118 (0.038)	0.100 (0.050)	−0.001 (0.029)

Variable importance values sum model weights across the entire data set for each variable. Unconditional model-averaged parameter estimates with associated standard errors are based on standardized values. Potential Evapotranspiration and Agriculture include both main effect (m) and quadratic (q) terms, whereas all other covariates report linear relationships.
