# Supplementary material for: Biotic and abiotic factors predicting the global distribution and population density of an invasive large mammal
Source: Sci Rep. 2017 Mar 9;7:44152. doi: 10.1038/srep44152 (PMC5343451; doi:10.1038/srep44152)
Supplement: Supplementary Files [file srep44152-s1.doc]

**Biotic and abiotic factors predicting the global distribution and population density of an invasive large mammal**

Jesse S. Lewis^1*^, Matthew L. Farnsworth^1^, Chris L. Burdett^2^, David M. Theobald^1^, Miranda Gray^3^, Ryan S. Miller^4^

Supplementary Files 1, 2, 3, 4, and 5

^1^ Conservation Science Partners, 5 Old Town Sq, Suite 205, Fort Collins, Colorado, USA 80524, jlewis@csp-inc.org, matthew.l.farnsworth@gmail.com, davet@csp-inc.org

^2^ Colorado State University, Department of Biology, Fort Collins, Colorado, USA 80524, chris.burdett@colostate.edu

^3^ Conservation Science Partners, c, Truckee, California, USA 96161, miranda@csp-inc.org

^4^ United States Department of Agriculture, Animal and Plant Health Inspection Service, Veterinary Services, Center for Epidemiology and Animal Health, Fort Collins, Colorado, USA 80524, ryan.s.miller@aphis.usda.gov

^*^ Corresponding author: Jesse S Lewis; jslewis.research@gmail.com; telephone (970) 484 – 2898

**Supplementary Methods S1.** Methods and studies used to create the global distribution map of wild pigs across large land areas within their native and non-native range (Figure 1).

**Methods**

We mapped the extent of occurrence of wild pigs by first compiling existing spatial datasets depicting *Sus scrofa*’s geographic range . Second, we created additional spatial data by digitizing range maps or known occurrence locations from publications. We paid particular attention to range boundaries and areas where their distribution was poorly understood. To upscale fine-grained spatial data digitized from publications into a similar resolution as the existing broad-scale range maps, we buffered point locations by 10 km and then intersected the buffered points or polygons with Pfafstetter Level 6 watersheds obtained from the HydroSHEDS spatial hydrography database . We used watersheds to spatially filter these data because they provide a biologically meaningful way to depict the presence of wild pigs at a landscape-scale resolution [^5^](#_ENREF_5). Lastly, we differentiated two categories of occurrence, areas where the presence of wild pigs has been confirmed through published maps or data, and other areas where the occurrence of wild pigs is less certain. All digitizing and map development was performed using ArcGIS software [^6^](#_ENREF_6).

**Studies used in constructing distribution**

*Global*

[Oliver and Brisbi^n 7^](#_ENREF_7), [Lon^g 8^](#_ENREF_8), [Meijaard, et al^. 9^](#_ENREF_9), [Barrios-Garcia and Ballar^i 10^](#_ENREF_10)

*Eurasia*

[Erkinaro, et al^. 11^](#_ENREF_11), [Oliver and Leu^s 12^](#_ENREF_12), [Campbell and Hartle^y 13^](#_ENREF_13), [Magnusso^n 14^](#_ENREF_14), [NBDC^I 15^](#_ENREF_15), [Haaverstad, et al^. 16^](#_ENREF_16), [IUC^N 2^](#_ENREF_2), [Ukkonen, et al^. 17^](#_ENREF_17), [Wilso^n 18^](#_ENREF_18)

*Africa*

[Blenc^h 19^](#_ENREF_19), [Phiri, et al^. 20^](#_ENREF_20), [Kisakye and Masab^a 21^](#_ENREF_21), [Pouedet, et al^. 22^](#_ENREF_22), [Ngowi, et al^. 23^](#_ENREF_23), [Githigia, et al^. 24^](#_ENREF_24), [Waiswa, et al^. 25^](#_ENREF_25), [Assana, et al^. 26^](#_ENREF_26), [Kingdon and Hoffman^n 27^](#_ENREF_27), [Thomas, et al^. 28^](#_ENREF_28), [Ouma, et al^. 29^](#_ENREF_29)

*Australia*

[Wes^t 30^](#_ENREF_30)

*United States and Canada*

[SCWD^S 31^](#_ENREF_31), Ruth Kost and Ryan Brook, University of Saskatchewan, personal communication

*Mexico*

[Álvarez-Romero, et al^. 32^](#_ENREF_32), [Solís-Cámara, et al^. 33^](#_ENREF_33), [Hidalgo-Mihart, et al^. 34^](#_ENREF_34)

*South America*

[Merino and Carpinett^i 35^](#_ENREF_35), [Merino, et al^. 36^](#_ENREF_36), [Desbiez, et al^. 37^](#_ENREF_37), [Desbiez, et al^. 38^](#_ENREF_38), [Salvador and Fernande^z 39^](#_ENREF_39), [Kaizer, et al^. 40^](#_ENREF_40), [Aravena, et al^. 41^](#_ENREF_41), [Ballari, et al^. 42^](#_ENREF_42), [Pedrosa, et al^. 43^](#_ENREF_43), [Skewes and Jaksi^c 44^](#_ENREF_44)

**References**

**Supplementary Table S2.** Studies used in analyses evaluating the relationship between wild pig population density and biotic and abiotic factors across Europe, Asia, Australia, North America, South America, and several islands. Location coordinates (x,y) are presented in decimal degrees.

| # | Continent | Country | Density (# / km^2^) | x | y | Reference |
| --- | --- | --- | --- | --- | --- | --- |
| 1 | Asia | India | 2.46 | 13.509 | 75.631 | [Gopalaswamy, et al^. 1^](#_ENREF_1) |
| 2 | Asia | Malaysia | 3.63 | 4.533 | 102.429 | [Kawanishi and Sunquis^t 2^](#_ENREF_2) |
| 3 | Asia | Pakistan | 3.70 | 24.538 | 67.959 | [Smiet, et al^. 3^](#_ENREF_3) |
| 4 | Asia | Nepal | 4.00 | 28.583 | 81.333 | [Dinerstei^n 4^](#_ENREF_4) |
| 5 | Asia | Malaysia | 4.17 | 4.623 | 102.068 | [Kawanishi and Sunquis^t 2^](#_ENREF_2) |
| 6 | Asia | India | 4.20 | 12.025 | 76.108 | [Karanth and Sunquis^t 5^](#_ENREF_5) |
| 7 | Asia | Malaysia | 4.62 | 4.847 | 102.450 | [Kawanishi and Sunquis^t 2^](#_ENREF_2) |
| 8 | Asia | Nepal | 5.80 | 27.551 | 84.471 | [Seidensticke^r 6^](#_ENREF_6) |
| 9 | Asia | Malaysia | 37.00 | 2.983 | 102.210 | [Icke^s 7^](#_ENREF_7) |
| 10 | Australia | Australia | 0.40 | -31.110 | 145.213 | [Choquenot, et al^. 8^](#_ENREF_8) |
| 11 | Australia | Australia | 0.89 | -35.500 | 148.999 | [Hon^e 9^](#_ENREF_9) |
| 12 | Australia | Australia | 1.01 | -28.336 | 150.675 | [Wilson, et al^. 10^](#_ENREF_10) |
| 13 | Australia | Australia | 1.60 | -36.718 | 148.530 | [Saunders and Gile^s 11^](#_ENREF_11) |
| 14 | Australia | Australia | 1.75 | -35.750 | 148.991 | [McIlroy, et al^. 12^](#_ENREF_12), [Hon^e 13^](#_ENREF_13) |
| 15 | Australia | Australia | 1.92 | -29.850 | 144.147 | [Choqueno^t 14^](#_ENREF_14), [Dexte^r 15^](#_ENREF_15) |
| 16 | Australia | Australia | 2.00 | -33.481 | 149.788 | [Saunders and Ka^y 16^](#_ENREF_16) |
| 17 | Australia | Australia | 2.40 | -29.838 | 145.358 | [Choquenot, et al^. 8^](#_ENREF_8) |
| 18 | Australia | Australia | 2.80 | -14.500 | 131.183 | [Cale^y 17^](#_ENREF_17) |
| 19 | Australia | Australia | 3.30 | -18.184 | 145.981 | [Mitchel^l 18^](#_ENREF_18) |
| 20 | Australia | Australia | 4.00 | -14.548 | 144.144 | [Mitchel^l 19^](#_ENREF_19) |
| 21 | Australia | Australia | 5.80 | -30.820 | 143.920 | [Choquenot, et al^. 8^](#_ENREF_8) |
| 22 | Australia | Australia | 10.00 | -31.006 | 147.569 | [Saunders and Bryan^t 20^](#_ENREF_20) |
| 23 | Europe | Russia | 0.01 | 56.347 | 44.012 | [Fadee^v 21^](#_ENREF_21) ^*^ |
| 24 | Europe | Russia | 0.02 | 53.163 | 45.074 | [Fadee^v 21^](#_ENREF_21) ^*^ |
| 25 | Europe | Russia | 0.02 | 53.999 | 44.000 | [Fadee^v 21^](#_ENREF_21) ^*^ |
| 26 | Europe | Russia | 0.02 | 57.020 | 41.068 | [Fadee^v 21^](#_ENREF_21) ^*^ |
| 27 | Europe | Russia | 0.03 | 56.164 | 40.506 | [Fadee^v 21^](#_ENREF_21) ^*^ |
| 28 | Europe | Russia | 0.03 | 56.633 | 59.850 | [Fadee^v 21^](#_ENREF_21) ^*^ |
| 29 | Europe | Russia | 0.03 | 60.000 | 31.000 | [Fadee^v 21^](#_ENREF_21) ^*^ |
| 30 | Europe | Russia | 0.04 | 57.000 | 39.000 | [Fadee^v 21^](#_ENREF_21) ^*^ |
| 31 | Europe | Russia | 0.04 | 57.500 | 61.000 | [Fadee^v 21^](#_ENREF_21) ^*^ |
| 32 | Europe | Poland | 0.05 | 49.383 | 22.420 | [Fonseca, et al^. 22^](#_ENREF_22) |
| 33 | Europe | Russia | 0.05 | 54.500 | 39.665 | [Fadee^v 21^](#_ENREF_21) ^*^ |
| 34 | Europe | Russia | 0.07 | 58.500 | 31.499 | [Fadee^v 21^](#_ENREF_21) ^*^ |
| 35 | Europe | Russia | 0.08 | 52.669 | 41.512 | [Fadee^v 21^](#_ENREF_21) ^*^ |
| 36 | Europe | Russia | 0.08 | 57.583 | 39.749 | [Fadee^v 21^](#_ENREF_21) ^*^ |
| 37 | Europe | Russia | 0.09 | 52.667 | 39.498 | [Fadee^v 21^](#_ENREF_21) ^*^ |
| 38 | Europe | Poland | 0.09 | 49.487 | 21.735 | [Fonseca, et al^. 22^](#_ENREF_22) |
| 39 | Europe | Russia | 0.11 | 54.167 | 37.500 | [Fadee^v 21^](#_ENREF_21) ^*^ |
| 40 | Europe | Russia | 0.11 | 57.000 | 36.000 | [Fadee^v 21^](#_ENREF_21) ^*^ |
| 41 | Europe | Belarus | 0.12 | 54.691 | 28.383 | [Lavo^v 23^](#_ENREF_23) ^*^ |
| 42 | Europe | Russia | 0.14 | 55.638 | 37.487 | [Fadee^v 21^](#_ENREF_21) ^*^ |
| 43 | Europe | Poland | 0.15 | 49.648 | 19.625 | [Fonseca, et al^. 22^](#_ENREF_22) |
| 44 | Europe | Russia | 0.16 | 51.703 | 39.216 | [Fadee^v 21^](#_ENREF_21) ^*^ |
| 45 | Europe | Russia | 0.16 | 54.667 | 32.000 | [Fadee^v 21^](#_ENREF_21) ^*^ |
| 46 | Europe | Kazakhstan | 0.18 | 43.510 | 72.483 | [Fedosenko and Zhiryako^v 24^](#_ENREF_24) ^*^ |
| 47 | Europe | Russia | 0.19 | 50.500 | 36.497 | [Fadee^v 21^](#_ENREF_21) ^*^ |
| 48 | Europe | Belarus | 0.19 | 52.517 | 26.992 | [Kozl^o 25^](#_ENREF_25) ^*^ |
| 49 | Europe | Russia | 0.20 | 54.500 | 36.750 | [Fadee^v 21^](#_ENREF_21) ^*^ |
| 50 | Europe | Russia | 0.21 | 58.000 | 28.500 | [Fadee^v 21^](#_ENREF_21) ^*^ |
| 51 | Europe | Russia | 0.22 | 58.750 | 37.500 | [Tupicin^a 26^](#_ENREF_26) ^*^ |
| 52 | Europe | Belarus | 0.32 | 53.666 | 28.987 | [Kozl^o 25^](#_ENREF_25) ^*^ |
| 53 | Europe | Russia | 0.35 | 53.167 | 34.500 | [Fadee^v 21^](#_ENREF_21) ^*^ |
| 54 | Europe | Poland | 0.37 | 50.082 | 20.377 | [Pucek, et al^. 27^](#_ENREF_27) ^*^ |
| 55 | Europe | Russia | 0.43 | 51.667 | 36.166 | [Fadee^v 21^](#_ENREF_21) ^*^ |
| 56 | Europe | Poland | 0.46 | 49.834 | 21.501 | [Fonseca, et al^. 22^](#_ENREF_22) |
| 57 | Europe | Poland | 0.48 | 49.116 | 22.729 | [Kanzaki, et al^. 28^](#_ENREF_28) ^*^ |
| 58 | Europe | Spain | 0.61 | 41.510 | -5.488 | [Tellería and Sáez-Royuel^a 29^](#_ENREF_29) ^*^ |
| 59 | Europe | Poland | 0.64 | 53.885 | 23.030 | [Fonseca, et al^. 22^](#_ENREF_22) |
| 60 | Europe | Belarus | 0.72 | 55.501 | 28.996 | [Kozl^o 25^](#_ENREF_25) ^*^ |
| 61 | Europe | Poland | 0.79 | 50.049 | 19.640 | [Fonseca, et al^. 22^](#_ENREF_22) |
| 62 | Europe | Poland | 0.88 | 53.833 | 23.289 | [Pucek, et al^. 30^](#_ENREF_30) ^*^ |
| 63 | Europe | Poland | 0.94 | 50.875 | 15.560 | [Fonseca, et al^. 22^](#_ENREF_22) |
| 64 | Europe | Poland | 1.06 | 54.103 | 22.277 | [Fonseca, et al^. 22^](#_ENREF_22) |
| 65 | Europe | Lithuania | 1.10 | 54.868 | 23.780 | [Janulaiti^s 31^](#_ENREF_31) ^*^ |
| 66 | Europe | Belarus | 1.16 | 52.707 | 24.006 | [Kozl^o 25^](#_ENREF_25) ^*^ |
| 67 | Europe | Poland | 1.26 | 53.610 | 21.549 | [Fonseca, et al^. 22^](#_ENREF_22) |
| 68 | Europe | Kazakhstan | 1.50 | 43.501 | 77.498 | [Fedosenko and Zhiryako^v 24^](#_ENREF_24) ^*^ |
| 69 | Europe | Poland | 1.58 | 50.182 | 19.527 | [Pucek, et al^. 30^](#_ENREF_30) ^*^ |
| 70 | Europe | Poland | 1.64 | 49.600 | 18.832 | [Pucek, et al^. 30^](#_ENREF_30) ^*^ |
| 71 | Europe | Italy | 1.70 | 44.500 | 8.999 | [Marsan, et al^. 32^](#_ENREF_32) ^*^ |
| 72 | Europe | Poland | 1.83 | 50.736 | 18.891 | [Fonseca, et al^. 22^](#_ENREF_22) |
| 73 | Europe | Poland | 1.89 | 50.375 | 22.197 | [Fonseca, et al^. 22^](#_ENREF_22) |
| 74 | Europe | Poland | 1.99 | 53.511 | 16.437 | [Fonseca, et al^. 22^](#_ENREF_22) |
| 75 | Europe | Poland | 2.02 | 52.546 | 17.115 | [Pucek, et al^. 30^](#_ENREF_30) ^*^ |
| 76 | Europe | Poland | 2.20 | 50.459 | 18.955 | [Fonseca, et al^. 22^](#_ENREF_22) |
| 77 | Europe | Poland | 2.21 | 51.408 | 15.442 | [Bobe^k 33^](#_ENREF_33) |
| 78 | Europe | Germany | 2.40 | 52.002 | 13.006 | [Kern, et al^. 34^](#_ENREF_34) ^*^ |
| 79 | Europe | France | 2.50 | 43.500 | 1.748 | [Spitz and Janea^u 35^](#_ENREF_35) ^*^ |
| 80 | Europe | Poland | 2.65 | 52.735 | 23.854 | [Melis, et al^. 36^](#_ENREF_36) |
| 81 | Europe | Poland | 2.67 | 50.527 | 16.707 | [Fonseca, et al^. 22^](#_ENREF_22) |
| 82 | Europe | France | 2.70 | 43.498 | 4.519 | [Dardaillo^n 37^](#_ENREF_37) ^*^ |
| 83 | Europe | Italy | 3.00 | 42.628 | 11.122 | [Massei, et al^. 38^](#_ENREF_38) ^*^ |
| 84 | Europe | Poland | 3.05 | 50.591 | 17.802 | [Fonseca, et al^. 22^](#_ENREF_22) |
| 85 | Europe | Spain | 3.10 | 42.500 | -0.997 | [Herrero, et al^. 39^](#_ENREF_39) ^*^ |
| 86 | Europe | Spain | 3.50 | 40.005 | -6.334 | [Fernández-Llario, et al^. 40^](#_ENREF_40) ^*^ |
| 87 | Europe | Poland | 3.55 | 54.659 | 18.236 | [Fonseca, et al^. 22^](#_ENREF_22) |
| 88 | Europe | Italy | 3.57 | 43.500 | 11.000 | [Monaco, et al^. 41^](#_ENREF_41) ^*^ |
| 89 | Europe | Azerbaijan | 3.59 | 38.921 | 48.849 | [Litvino^v 42^](#_ENREF_42) ^*^ |
| 90 | Europe | Poland | 3.59 | 52.963 | 15.607 | [Fonseca, et al^. 22^](#_ENREF_22) |
| 91 | Europe | Germany | 4.75 | 49.234 | 7.799 | [Ebert, et al^. 43^](#_ENREF_43) |
| 92 | Europe | Netherlands | 4.80 | 52.000 | 5.339 | [Kuiters and Sli^m 44^](#_ENREF_44) ^*^ |
| 93 | Europe | Italy | 6.20 | 43.800 | 11.817 | [Mattioli, et al^. 45^](#_ENREF_45) ^*^ |
| 94 | Europe | Czech Republic | 6.39 | 49.349 | 16.875 | [Plhal, et al^. 46^](#_ENREF_46), [Plhal, et al^. 47^](#_ENREF_47) |
| 95 | Europe | Swedan | 7.50 | 58.972 | 17.534 | [Welande^r 48^](#_ENREF_48) |
| 96 | Europe | Italy | 9.59 | 41.708 | 12.403 | [Focardi, et al^. 49^](#_ENREF_49), [Focardi, et al^. 50^](#_ENREF_50) |
| 97 | Europe | Italy | 9.78 | 43.132 | 11.166 | [Boitani, et al^. 51^](#_ENREF_51) ^*^ |
| 98 | Europe | Spain | 10.00 | 37.009 | -6.479 | [Fernández-Llario, et al^. 40^](#_ENREF_40) ^*^ |
| 99 | Europe | Switzerland | 10.35 | 46.185 | 6.021 | [Hebeisen, et al^. 52^](#_ENREF_52) |
| 100 | Europe | Poland | 12.07 | 53.297 | 14.712 | [Fonseca, et al^. 22^](#_ENREF_22) |
| 101 | North America | USA | 0.65 | 30.696 | -104.094 | [Adkins and Harvesto^n 53^](#_ENREF_53) |
| 102 | North America | USA | 1.00 | 38.996 | -123.367 | [Sweitzer, et al^. 54^](#_ENREF_54) |
| 103 | North America | USA | 1.10 | 35.972 | -121.233 | [Pine and Gerde^s 55^](#_ENREF_55) |
| 104 | North America | USA | 1.20 | 38.713 | -123.000 | [Sweitzer, et al^. 54^](#_ENREF_54) |
| 105 | North America | USA | 1.30 | 36.487 | -121.854 | [Sweitzer, et al^. 54^](#_ENREF_54) |
| 106 | North America | USA | 1.90 | 38.537 | -123.007 | [Sweitzer, et al^. 54^](#_ENREF_54) |
| 107 | North America | USA | 1.90 | 35.662 | -120.800 | [Sweitzer, et al^. 56^](#_ENREF_56) |
| 108 | North America | USA | 2.37 | 33.146 | -81.685 | [Kigh^t 57^](#_ENREF_57), [Sweene^y 58^](#_ENREF_58), [Crouc^h 59^](#_ENREF_59) |
| 109 | North America | USA | 2.80 | 28.326 | -99.429 | [Gabor, et al^. 60^](#_ENREF_60) |
| 110 | North America | USA | 3.80 | 37.169 | -121.421 | [Sweitzer, et al^. 54^](#_ENREF_54) |
| 111 | North America | USA | 3.80 | 37.349 | -121.641 | [Schauss, et al^. 61^](#_ENREF_61) |
| 112 | North America | USA | 4.85 | 35.584 | -83.740 | [Singe^r 62^](#_ENREF_62) |
| 113 | North America | USA | 5.51 | 40.104 | -121.959 | [Patte^n 63^](#_ENREF_63), [Barret^t 64^](#_ENREF_64) |
| 114 | North America | USA | 6.13 | 32.405 | -84.729 | [Hanson, et al^. 65^](#_ENREF_65) |
| 115 | North America | USA | 7.00 | 28.675 | -80.737 | [Singe^r 62^](#_ENREF_62) |
| 116 | North America | USA | 9.50 | 28.121 | -97.376 | [Ilse and Hellgre^n 66^](#_ENREF_66) |
| 117 | South America | Argentina | 3.00 | -36.343 | -57.278 | [Merino and Carpinett^i 67^](#_ENREF_67), [Pérez Carusi, et al^. 68^](#_ENREF_68) |
| 118 | South America | Brazil | 5.03 | -18.999 | -56.663 | [Desbiez, et al^. 69^](#_ENREF_69) |
| 119 | Island | Sri Lanka | 0.90 | 7.577 | 80.765 | [Eisenberg and Lockhar^t 70^](#_ENREF_70), [McKa^y 71^](#_ENREF_71), [Santiapillai and Chamber^s 72^](#_ENREF_72) |
| 120 | Island | Australia (Flinders Island, Tasmania) | 1.77 | -39.985 | 148.085 | [Statham and Middleto^n 73^](#_ENREF_73) |
| 121 | Island | Indonesia (Sumatra) | 5.02 | -5.250 | 104.137 | [O'Brien, et al^. 74^](#_ENREF_74) |
| 122 | Island | USA (Hawaii) | 5.13 | 19.457 | -155.288 | [Anderson and Ston^e 75^](#_ENREF_75), [Scheffler, et al^. 76^](#_ENREF_76) |
| 123 | Island | USA (Hawaii) | 12.36 | 20.713 | -156.099 | [Dion^g 77^](#_ENREF_77), [Anderson and Ston^e 78^](#_ENREF_78) |
| 124 | Island | Ecuador (Galapagos) | 26.00 | -0.263 | -90.747 | [Coblentz and Babe^r 79^](#_ENREF_79) |
| 125 | Island | Japan | 26.80 | 35.237 | 140.092 | [Osada, et al^. 80^](#_ENREF_80) |
| 126 | Island | New Zealand | 27.75 | -41.793 | 172.418 | [McIlro^y 81^](#_ENREF_81) |
| 127 | Island | USA (Santa Catalina) | 28.00 | 33.357 | -118.422 | [Baber and Coblent^z 82^](#_ENREF_82) |
| 128 | Island | Indonesia (Java) | 29.50 | -6.741 | 105.257 | [Pauwel^s 83^](#_ENREF_83) |
| 129 | Island | USA (Santa Cruz) | 40.45 | 34.005 | -119.766 | [Sterner and Barret^t 84^](#_ENREF_84), [Parkes, et al^. 85^](#_ENREF_85) |

Notes: ^*^ cited in [Melis, et al^. 36^](#_ENREF_36)

**Literature Cited**

1 Gopalaswamy, A., Karanth, K., Kumar, N. & Macdonald, D. Estimating tropical forest ungulate densities from sign surveys using abundance models of occupancy. *Animal Conservation* **15**, 669-679 (2012).

2 Kawanishi, K. & Sunquist, M. E. Conservation status of tigers in a primary rainforest of Peninsular Malaysia. *Biological Conservation* **120**, 329-344 (2004).

3 Smiet, A., Fulk, G. & Lathiya, S. Wild boar ecology in Thatta district: a preliminary study. *Pak. J. Zool* **11**, 295-302 (1979).

4 Dinerstein, E. An ecological survey of the Royal Karnali-Bardia Wildlife Reserve, Nepal: Part III: Ungulate populations. *Biological Conservation* **18**, 5-37 (1980).

5 Karanth, K. U. & Sunquist, M. E. Population structure, density and biomass of large herbivores in the tropical forests of Nagarahole, India. *Journal of Tropical Ecology* **8**, 21-35 (1992).

6 Seidensticker, J. Ungulate populations in Chitawan valley, Nepal. *Biological Conservation* **10**, 183-210 (1976).

7 Ickes, K. Hyper-abundance of native wild pigs (Sus scrofa) in a lowland Dipterocarp rain forest of peninsular Malaysia *Biotropica* **33**, 682-690 (2001).

8 Choquenot, D., Lukins, B. & Curran, G. Assessing lamb predation by feral pigs in Australia's semi-arid rangelands. *Journal of Applied Ecology* **34**, 1445-1454 (1997).

9 Hone, J. Evaluation of methods for ground survey of feral pigs and their sign. *Acta Theriologica* **33**, 451-465 (1988).

10 Wilson, G., Hill, G. & Barnes, A. An aerial survey of feral pigs and emus in southeastern Queensland. *Wildlife Research* **14**, 515-520 (1987).

11 Saunders, G. & Giles, J. Ecological comparison of two wild pig populations in semi-arid and sub-alpine Australia. *Journal of Mountain Ecology* **3**, 152-155 (1995).

12 McIlroy, J., Braysher, M. & Saunders, G. Effectiveness of a warfarin-poisoning campaign against feral pigs, Sus scrofa, in Namadgi National Park, ACT. *Wildlife Research* **16**, 195-202 (1989).

13 Hone, J. Feral pigs in Namadgi National Park, Australia: dynamics, impacts and management. *Biological Conservation* **105**, 231-242 (2002).

14 Choquenot, D. *The dynamics of feral pig populations in the semi-arid rangelands of eastern Australia* Ph.D. thesis, University of Sydney, (1994).

15 Dexter, N. *The behaviour of feral pigs in north-west New South Wales and its implications for the epidemiology of foot and mouth disease* Ph.D. thesis, University of New England, (1996).

16 Saunders, G. & Kay, B. Movements of feral pigs (Sus scrofa) at Sunny Corner, New South Wales. *Wildlife Research* **18**, 49-61 (1991).

17 Caley, P. Population dynamics of feral pigs (Sus scrofa) in a tropical riverine habitat complex. *Wildlife Research* **20**, 625-636 (1993).

18 Mitchell, J. L. *Ecology and management of feral pigs (Sus scrofa) in rainforests* Ph.D. thesis, James Cook University, (2002).

19 Mitchell, J. The effectiveness of aerial baiting for control of feral pigs (Sus scrofa) in North Queensland. *Wildlife Research* **25**, 297-303 (1998).

20 Saunders, G. & Bryant, H. The evaluation of a feral pig eradication program during a simulated exotic disease outbreak. *Wildlife Research* **15**, 73-81 (1988).

21 Fadeev, E. Population dynamics of wild boar (Sus scrofa) in European Russia. *Zoologicheskii Zhurnal* **52**, 1214-1219 (1973).

22 Fonseca, C., Kolecki, M., Merta, D. & Bobek, B. Use of line intercept track index and plot sampling for estimating wild boar, Sus scrofa (Suidae), densities in Poland. *Folia Zoologica* **56**, 389-398 (2007).

23 Lavov, M. Dinamika i regulirovanie chislennosti kabana w Berezinskom zapovednike [Dynamics and regulation of wild boar abundance in the Berezinskii Reserve]. *Zapovedniki Belarusii*, 93-98 (1981).

24 Fedosenko, A. & Zhiryakov, V. Ekologiya i povedene kabana v gorakh yuga i yugo-vostoka Kazakhstana.[Ecology and behaviour of the wild boar in the southern and southeastern Kazakhstan]. *Byulleten Moskovskogo Obshchestva Ispytatelei Prirody, Otdelenie Biologii* **89**, 36-45 (1984).

25 Kozlo, P. Factors determining the population dynamics of wild boar in Belovezhskyi forest. *Zoologicheskii Zhurnal* **49**, 422-430 (1970).

26 Tupicina, L. Dinamika chislennosti i razmeshchenie kabana (Sus scrofa) v Darvinskom Zapovednike [Population dynamics and distribution of wild boar (Sus scrofa) in the Darvinskii Reserve]. *Populyacionnye issledovanya zhivotnykh v zapovednikakh [Populational research in reserves]*, 128-139 (1988).

27 Pucek, Z., Jędrzejewski, W., Jędrzejewska, B. & Pucek, M. Rodent population dynamics in a primeval deciduous forest (Białowieża National Park) in relation to weather, seed crop, and predation. *Acta Theriologica* **38**, 199-232 (1993).

28 Kanzaki, N., Perzanowski, K. & Nowosad, M. Factors affecting wild boar (Sus scrofa) population dynamics in Bieszczady, Poland. *Gibier faune sauvage* **15**, 1171-1178 (1998).

29 Tellería, J. & Sáez-Royuela, C. Ecología de una población ibérica de lobos (Canis lupus). . *Acta Vertebrata Doñana* **16**, 105-122 (1989).

30 Pucek, Z. *et al.* Estimates of density and number of ungulates. *Polish Ecological Studies* **1**, 121-135 (1975).

31 Janulaitis, Z. Distribution, abundance and regulation of wild boar population in Lithuania. *Acta Zoologica Lituanica* **13**, 88-88 (2003).

32 Marsan, A., Spano, S. & Tognoni, C. Management attempts of wild boar (Sus scrofa L.): first results and outstanding researches in northern Apennines (Italy). *Journal of Mountain Ecology* **3**, 219–221 (1995).

33 Bobek, B. Use of a line intercept snow track index and plot sampling for estimating densities of wild boar (Sus scrofa) in southwestern Poland. *Wildlife Biology in Practice* **10**, 7-16 (2014).

34 Kern, B. *et al.* Incidence of classical swine fever (CSF) in wild boar in a densely populated area indicating CSF virus persistence as a mechanism for virus perpetuation. *Journal of Veterinary Medicine, Series B* **46**, 63-68 (1999).

35 Spitz, F. & Janeau, G. Spatial strategies: an attempt to classify daily movements of wild boar. *Acta Theriologica* **35**, 129-149 (1990).

36 Melis, C., Szafrańska, P. A., Jędrzejewska, B. & Bartoń, K. Biogeographical variation in the population density of wild boar (Sus scrofa) in western Eurasia. *Journal of Biogeography* **33**, 803-811 (2006).

37 Dardaillon, M. Seasonal variations in habitat selection and spatial distribution of wild boar (Sus scrofa) in the Camargue, Southern France. *Behavioural Processes* **13**, 251-268 (1986).

38 Massei, G., Genov, P., Staines, B. & Gorman, M. Mortality of wild boar, Sus scrofa, in a Mediterranean area in relation to sex and age. *Journal of Zoology* **242**, 394-400 (1997).

39 Herrero, J., García-Serrano, A. & García-González, R. Wild boar (Sus scrofa L.) hunting in south-western Pyrenees (Spain): preliminary data. *Journal of Mountain Ecology* **3**, 228-229 (1995).

40 Fernández-Llario, P., Mateos-Quesada, P., Silverio, A. & Santos, P. Habitat effects and shooting techniques on two wild boar (Sus scrofa) populations in Spain and Portugal. *Zeitschrift für Jagdwissenschaft* **49**, 120-129 (2003).

41 Monaco, A., Pedrotti, L. & Franzetti, B. in *24th Congress of International Union of Game Biologists, Thessaloniki, Greece.* 87.

42 Litvinov, V. Vliyanie volka na chislennost kabana v Kyzyl-Agachskom zapovednike [Influence of wolf on wild boar abundance in the Kyzyl-Agach Reserve]. *Kopytnye fauny USSR [Ungulate fauna of the USSR]*, 173-175 (1980).

43 Ebert, C., Knauer, F., Spielberger, B., Thiele, B. & Hohmann, U. Estimating wild boar Sus scrofa population size using faecal DNA and capture-recapture modelling. *Wildlife Biology* **18**, 142-152 (2012).

44 Kuiters, A. & Slim, P. Regeneration of mixed deciduous forest in a Dutch forest-heathland, following a reduction of ungulate densities. *Biological Conservation* **105**, 65-74 (2002).

45 Mattioli, L., Apollonio, M., Mazzarone, V. & Centofanti, E. Wolf food habits and wild ungulate availability in the Foreste Casentinesi National Park, Italy. *Acta Theriologica* **40**, 387-402 (1995).

46 Plhal, R., Kamler, J., Homolka, M. & Adamec, Z. An assessment of the applicability of photo trapping to estimate wild boar population density in a forest environment. *Folia Zoologica* **60**, 237-246 (2011).

47 Plhal, R., Kamler, J. & Homolka, M. Faecal pellet group counting as a promising method of wild boar population density estimation. *Acta Theriologica* **59**, 561-569 (2014).

48 Welander, J. Are wild boars a future threat to the Swedish flora? *Journal of Mountain Ecology* **3**, 165-167 (1995).

49 Focardi, S., Isotti, R., Pelliccioni, E. R. & Iannuzzo, D. The use of distance sampling and mark-resight to estimate the local density of wildlife populations. *Environmetrics* **13**, 177-186 (2002).

50 Focardi, S., Isotti, R. & Tinelli, A. Line transect estimates of ungulate populations in a Mediterranean forest. *The Journal of Wildlife Management* **66**, 48-58 (2002).

51 Boitani, L., Trapanese, P. & Mattei, L. Methods of population estimates of a hunted wild boar (Sus scrofa L.) population in Tuscany (Italy). *Journal of Mountain Ecology* **3**, 204-208 (1995).

52 Hebeisen, C., Fattebert, J., Baubet, E. & Fischer, C. Estimating wild boar (Sus scrofa) abundance and density using capture–resights in Canton of Geneva, Switzerland. *European Journal of Wildlife Research* **54**, 391-401 (2008).

53 Adkins, R. N. & Harveston, L. A. Demographic and spatial characteristics of feral hogs in the Chihuahuan Desert, Texas. *Human-Wildlife Interactions* **1**, 152-160 (2007).

54 Sweitzer, R. A., Van Vuren, D., Gardner, I. A., Boyce, W. M. & Waithman, J. D. Estimating sizes of wild pig populations in the north and central coast regions of California. *The Journal of wildlife management* **64**, 531-543 (2000).

55 Pine, D. & Gerdes, G. Wild pigs in Monterey County, California. *California Fish and Game* **59**, 126-137 (1973).

56 Sweitzer, R. A., Gardner, I. A., Gonzales, B. J., Van Vuren, D. & Boyce, W. M. Population densities and disease surveys of wild pigs in the coast ranges of central and northern California. *Proceedings of 17th Vertebrate Pest Conference*, 75-82 (1996).

57 Kight, J. *An ecological study of the bobcat, Lynx rufus (Schreber), in west-central South Carolina*, University of Georgia, Athens, GA, USA, (1962).

58 Sweeney, J. *Preliminary investigations of a feral hog population on the Savannah River Plant, South Carolina*, M.S. Thesis, University of Georgia, Athens, Georgia, USA, (1970).

59 Crouch, L. C. *Movements of and habitat utilization by feral hogs at the Savannah River Plant, South Carolina* M.S. Thesis thesis, Clemson University, Clemson, SC, USA, (1983).

60 Gabor, T. M., Hellgren, E. C., Bussche, R. A. & Silvy, N. J. Demography, sociospatial behaviour and genetics of feral pigs (Sus scrofa) in a semi-arid environment. *Journal of Zoology* **247**, 311-322 (1999).

61 Schauss, M. E., Coletto, H. J. & Kutilek, M. Population characteristics of wild pigs, Sus scrofa, in eastern Santa Clara County, California. *California Fish and Game* **76**, 68-77 (1990).

62 Singer, F. J. Wild pig populations in the national parks. *Environmental Management* **5**, 263-270 (1981).

63 Patten, D. C. Feral hogs - boon or burden. *Proceedings of the Sixth Vertebrate Pest Conference* **6**, 210-234 (1974).

64 Barrett, R. H. The feral hog on the Dye Creek Ranch, California. *Hilgardia* **46**, 283-355 (1978).

65 Hanson, L. B. *et al.* Change-in-ratio density estimator for feral pigs is less biased than closed mark–recapture estimates. *Wildlife Research* **35**, 695-699 (2008).

66 Ilse, L. M. & Hellgren, E. C. Spatial use and group dynamics of sympatric collared peccaries and feral hogs in southern Texas. *Journal of Mammalogy* **76**, 993-1002 (1995).

67 Merino, M. L. & Carpinetti, B. N. Feral pig Sus scrofa population estimates in Bahía Samborombón conservation area, Buenos Aires province, Argentina. *Journal of Neotropical Mammal* **10**, 269-275 (2003).

68 Pérez Carusi, L. C. *et al.* Relaciones espaciales y numéricas entre venados de las pampas (Ozotoceros bezoarticus celer) y chanchos cimarrones (Sus scrofa) en el Refugio de Vida Silvestre Bahía Samborombón, Argentina. *Ecología Austral* **19**, 63-71 (2009).

69 Desbiez, A. L. J., Keuroghlian, A., Piovezan, U. & Bodmer, R. E. Invasive species and bushmeat hunting contributing to wildlife conservation: the case of feral pigs in a Neotropical wetland. *Oryx* **45**, 78-83 (2011).

70 Eisenberg, J. F. & Lockhart, M. An ecological reconnaissance of Wilpattu National Park, Ceylon. (Smithsonian Institution Press Washington DC, USA, 1972).

71 McKay, G. M. Behavior and ecology of the Asiatic elephant in southeastern Ceylon. (Smithsonian Institution Press Washington, DC, USA, 1973).

72 Santiapillai, C. & Chambers, M. Aspects of the Population Dynamics of the Wild Pig (Sus scrofa Linnaeus, 1758) in the Ruhuna National Park. *Spixiana* **3**, 239-250 (1980).

73 Statham, M. & Middleton, M. in *Papers and proceedings of the Royal Society of Tasmania.* 121-124.

74 O'Brien, T. G., Kinnaird, M. F. & Wibisono, H. T. Crouching tigers, hidden prey: Sumatran tiger and prey populations in a tropical forest landscape. *Animal Conservation* **6**, 131-139 (2003).

75 Anderson, S. J. & Stone, C. P. Indexing sizes of feral pig populations in a variety of Hawaiian natural areas. *Transactions of the Western Section of the Wildlife Society* **30**, 26-39 (1994).

76 Scheffler, P. Y., Pratt, L. W., Foote, D. & Magnacca, K. N. A preliminary study of effects of feral pig density on native Hawaiian montane rainforest vegetation. *Technical Report 182* (2012).

77 Diong, C. H. *Population biology and management of the feral pig (Sus scrofa L.) in Kipahulu Valley, Maui* Ph.D. Thesis thesis, (1982).

78 Anderson, S. J. & Stone, C. P. Snaring to control feral pigs Sus scrofa in a remote Hawaiian rain forest. *Biological Conservation* **63**, 195-201 (1993).

79 Coblentz, B. & Baber, D. Biology and control of feral pigs on Isla Santiago, Galapagos, Ecuador. *Journal of Applied Ecology* **24**, 403-418 (1987).

80 Osada, Y., Kuriyama, T., Asada, M., Yokomizo, H. & Miyashita, T. Exploring the drivers of wildlife population dynamics from insufficient data by Bayesian model averaging. *Population Ecology* **57**, 485-493 (2015).

81 McIlroy, J. Aspects of the ecology of feral pigs (Sus scrofa) in the Murchison area, New Zealand. *New Zealand Journal of Ecology* **12**, 11-22 (1989).

82 Baber, D. W. & Coblentz, B. E. Density, home range, habitat use, and reproduction in feral pigs on Santa Catalina Island. *Journal of Mammalogy* **67**, 512-525 (1986).

83 Pauwels, W. *Study of Sus scrofa vittatus, its ecology and behavior in Ujung Kulon Nature Reserve, Java, Indonesia*, (1980).

84 Sterner, J. D. & Barrett, R. H. Removing feral pigs from Santa Cruz Island, California. *Transactions of the Western Section of the Wildlife Society* **27**, 47-53 (1991).

85 Parkes, J. P. *et al.* Rapid eradication of feral pigs (Sus scrofa) from Santa Cruz Island, California. *Biological Conservation* **143**, 634-641 (2010).

**Supplementary Figure S3.** Boxplot (including the median, first and third quartiles, minimum and maximum values, and outliers) demonstrating that population density (natural log scale; #/km^2^) of wild pigs is greater for island (n = 11) compared to mainland (n = 118) populations.


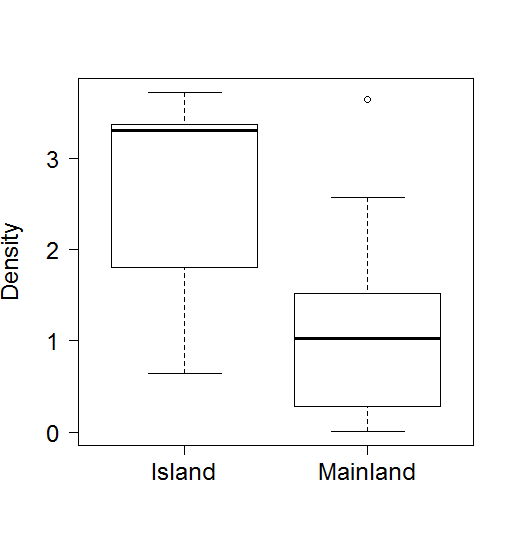


**Supplementary Table S4.** Model selection results using Akaike Information Criteria (AIC_c_) for all possible models evaluating how biotic and abiotic factors influenced population density of wild pigs. A “*” in the covariate columns indicates whether the variable was included in the model. K is the number of variables included in the model. Note that Potential Evapotranspiration and Agriculture include both main and quadratic effects (thus accounting for two parameters for each of these variables).

| # | Potential Evapotranspiration | Large Carnivore | Precipitation Wet Season | Unvegetated | Agriculture | Precipitation Dry Season | Forest | K | AIC_c_ | Δ AIC_c_ | weight | log(L) |
| --- | --- | --- | --- | --- | --- | --- | --- | --- | --- | --- | --- | --- |
| 1 | * | * | * | * | * | * |  | 10 | 237.94 | 0.00 | 0.68 | -108.33 |
| 2 | * | * | * | * | * | * | * | 11 | 240.18 | 2.24 | 0.22 | -108.32 |
| 3 | * | * | * | * | * |  |  | 9 | 243.00 | 5.06 | 0.05 | -111.98 |
| 4 | * | * | * | * | * |  | * | 10 | 244.40 | 6.46 | 0.03 | -111.56 |
| 5 | * | * | * | * |  | * |  | 8 | 246.14 | 8.20 | 0.01 | -114.65 |
| 6 | * | * | * | * |  | * | * | 9 | 248.20 | 10.26 | 0.00 | -114.58 |
| 7 | * | * | * |  | * | * |  | 9 | 248.25 | 10.31 | 0.00 | -114.60 |
| 8 | * | * | * |  | * | * | * | 10 | 249.14 | 11.20 | 0.00 | -113.93 |
| 9 | * | * | * |  | * |  | * | 9 | 252.95 | 15.01 | 0.00 | -116.95 |
| 10 | * | * | * | * |  |  |  | 7 | 253.93 | 15.99 | 0.00 | -119.64 |
| 11 | * | * |  | * | * | * |  | 9 | 254.00 | 16.06 | 0.00 | -117.48 |
| 12 | * | * | * | * |  |  | * | 8 | 255.58 | 17.64 | 0.00 | -119.37 |
| 13 | * | * |  | * | * | * | * | 10 | 256.16 | 18.22 | 0.00 | -117.44 |
| 14 | * | * | * |  | * |  |  | 8 | 256.35 | 18.41 | 0.00 | -119.76 |
| 15 | * | * | * |  |  | * |  | 7 | 256.47 | 18.53 | 0.00 | -120.91 |
| 16 | * | * | * |  |  | * | * | 8 | 258.28 | 20.34 | 0.00 | -120.72 |
| 17 | * | * |  | * |  | * |  | 7 | 263.05 | 25.11 | 0.00 | -124.20 |
| 18 | * | * |  | * | * |  | * | 9 | 264.78 | 26.84 | 0.00 | -122.87 |
| 19 | * | * |  | * |  | * | * | 8 | 265.17 | 27.23 | 0.00 | -124.17 |
| 20 | * | * |  | * | * |  |  | 8 | 265.82 | 27.88 | 0.00 | -124.49 |
| 21 | * | * | * |  |  |  | * | 7 | 266.28 | 28.34 | 0.00 | -125.82 |
| 22 | * |  |  | * | * | * |  | 8 | 266.61 | 28.67 | 0.00 | -124.89 |
| 23 | * |  | * | * | * | * |  | 9 | 267.29 | 29.35 | 0.00 | -124.12 |
| 24 | * | * | * |  |  |  |  | 6 | 267.91 | 29.97 | 0.00 | -127.71 |
| 25 | * |  |  | * | * | * | * | 9 | 268.17 | 30.23 | 0.00 | -124.56 |
| 26 | * |  | * | * | * | * | * | 10 | 268.23 | 30.29 | 0.00 | -123.47 |
| 27 | * |  | * | * | * |  |  | 8 | 273.12 | 35.18 | 0.00 | -128.14 |
| 28 | * |  |  | * | * |  |  | 7 | 275.08 | 37.14 | 0.00 | -130.22 |
| 29 | * |  | * | * | * |  | * | 9 | 275.33 | 37.39 | 0.00 | -128.14 |
| 30 | * |  |  | * | * |  | * | 8 | 276.81 | 38.87 | 0.00 | -129.99 |
| 31 | * | * |  | * |  |  | * | 7 | 277.80 | 39.86 | 0.00 | -131.58 |
| 32 | * | * |  | * |  |  |  | 6 | 279.16 | 41.22 | 0.00 | -133.34 |
| 33 | * |  |  | * |  | * |  | 6 | 282.40 | 44.46 | 0.00 | -134.96 |
| 34 | * |  |  | * |  | * | * | 7 | 282.86 | 44.92 | 0.00 | -134.11 |
| 35 | * |  | * | * |  | * | * | 8 | 283.64 | 45.70 | 0.00 | -133.41 |
| 36 | * |  | * | * |  | * |  | 7 | 284.04 | 46.10 | 0.00 | -134.70 |
| 37 | * | * |  |  | * | * | * | 9 | 285.98 | 48.04 | 0.00 | -133.47 |
| 38 |  | * | * |  | * | * |  | 7 | 289.55 | 51.61 | 0.00 | -137.45 |
| 39 |  | * | * |  | * | * | * | 8 | 290.88 | 52.94 | 0.00 | -137.03 |
| 40 | * |  | * |  | * | * |  | 8 | 291.27 | 53.33 | 0.00 | -137.22 |
| 41 |  | * | * | * | * | * |  | 8 | 291.73 | 53.79 | 0.00 | -137.45 |
| 42 |  | * | * |  | * |  |  | 6 | 291.82 | 53.88 | 0.00 | -139.67 |
| 43 |  | * | * | * | * | * | * | 9 | 292.53 | 54.59 | 0.00 | -136.74 |
| 44 | * |  | * |  | * | * | * | 9 | 293.09 | 55.15 | 0.00 | -137.03 |
| 45 |  | * | * | * | * |  |  | 7 | 293.56 | 55.62 | 0.00 | -139.46 |
| 46 | * | * |  |  | * | * |  | 8 | 293.91 | 55.97 | 0.00 | -138.54 |
| 47 | * |  | * | * |  |  |  | 6 | 293.94 | 56.00 | 0.00 | -140.73 |
| 48 |  | * | * |  | * |  | * | 7 | 293.95 | 56.01 | 0.00 | -139.65 |
| 49 | * |  |  | * |  |  |  | 5 | 294.52 | 56.58 | 0.00 | -142.09 |
| 50 |  | * | * | * | * |  | * | 8 | 295.66 | 57.72 | 0.00 | -139.41 |
| 51 | * |  | * | * |  |  | * | 7 | 295.98 | 58.04 | 0.00 | -140.67 |
| 52 | * |  |  | * |  |  | * | 6 | 296.57 | 58.63 | 0.00 | -142.04 |
| 53 | * | * |  |  | * |  | * | 8 | 296.69 | 58.75 | 0.00 | -139.93 |
| 54 |  | * | * |  |  | * |  | 5 | 296.84 | 58.90 | 0.00 | -143.25 |
| 55 | * | * |  |  |  | * | * | 7 | 297.38 | 59.44 | 0.00 | -141.37 |
| 56 |  | * | * | * |  | * | * | 7 | 297.84 | 59.90 | 0.00 | -141.60 |
| 57 |  | * | * |  |  | * | * | 6 | 298.03 | 60.09 | 0.00 | -142.78 |
| 58 |  | * | * | * |  | * |  | 6 | 298.20 | 60.26 | 0.00 | -142.86 |
| 59 | * |  | * |  | * |  | * | 8 | 300.38 | 62.44 | 0.00 | -141.78 |
| 60 | * |  |  |  | * | * | * | 8 | 302.10 | 64.16 | 0.00 | -142.63 |
| 61 |  | * | * | * |  |  |  | 5 | 302.23 | 64.29 | 0.00 | -145.94 |
| 62 | * |  | * |  | * |  |  | 7 | 302.41 | 64.47 | 0.00 | -143.89 |
| 63 | * | * |  |  |  | * |  | 6 | 302.56 | 64.62 | 0.00 | -145.04 |
| 64 |  | * | * |  |  |  |  | 4 | 303.12 | 65.18 | 0.00 | -147.45 |
| 65 |  | * | * | * |  |  | * | 6 | 304.03 | 66.09 | 0.00 | -145.77 |
| 66 | * |  |  |  | * | * |  | 7 | 304.35 | 66.41 | 0.00 | -144.85 |
| 67 |  | * | * |  |  |  | * | 5 | 305.03 | 67.09 | 0.00 | -147.34 |
| 68 | * |  | * |  |  | * |  | 6 | 311.30 | 73.36 | 0.00 | -149.41 |
| 69 | * |  |  |  | * |  | * | 7 | 313.10 | 75.16 | 0.00 | -149.23 |
| 70 | * |  | * |  |  | * | * | 7 | 313.39 | 75.45 | 0.00 | -149.37 |
| 71 | * | * |  |  |  |  | * | 6 | 315.66 | 77.72 | 0.00 | -151.59 |
| 72 | * |  |  |  |  | * |  | 5 | 320.24 | 82.30 | 0.00 | -154.95 |
| 73 | * |  |  |  |  | * | * | 6 | 321.06 | 83.12 | 0.00 | -154.29 |
| 74 | * | * |  |  | * |  |  | 7 | 327.15 | 89.21 | 0.00 | -156.25 |
| 75 | * |  | * |  |  |  |  | 5 | 328.80 | 90.86 | 0.00 | -159.23 |
| 76 | * |  | * |  |  |  | * | 6 | 329.10 | 91.16 | 0.00 | -158.31 |
| 77 | * |  |  |  | * |  |  | 6 | 332.32 | 94.38 | 0.00 | -159.92 |
| 78 | * |  |  |  |  |  | * | 5 | 341.70 | 103.76 | 0.00 | -165.68 |
| 79 |  |  | * | * | * | * | * | 8 | 342.80 | 104.86 | 0.00 | -162.98 |
| 80 | * | * |  |  |  |  |  | 5 | 343.29 | 105.35 | 0.00 | -166.47 |
| 81 |  |  | * |  | * | * | * | 7 | 346.89 | 108.95 | 0.00 | -166.12 |
| 82 |  |  | * |  | * | * |  | 6 | 348.35 | 110.41 | 0.00 | -167.94 |
| 83 |  |  | * | * | * | * |  | 7 | 349.39 | 111.45 | 0.00 | -167.37 |
| 84 |  |  | * | * | * |  | * | 7 | 349.84 | 111.90 | 0.00 | -167.60 |
| 85 |  |  | * | * | * |  |  | 6 | 351.44 | 113.50 | 0.00 | -169.48 |
| 86 |  |  | * |  | * |  |  | 5 | 352.13 | 114.19 | 0.00 | -170.89 |
| 87 |  |  | * |  | * |  | * | 6 | 353.94 | 116.00 | 0.00 | -170.73 |
| 88 | * |  |  |  |  |  |  | 4 | 354.89 | 116.95 | 0.00 | -173.33 |
| 89 |  |  | * | * |  | * | * | 6 | 355.73 | 117.79 | 0.00 | -171.62 |
| 90 |  | * |  | * | * | * |  | 7 | 356.29 | 118.35 | 0.00 | -170.82 |
| 91 |  | * |  | * | * | * | * | 8 | 357.46 | 119.52 | 0.00 | -170.31 |
| 92 |  | * |  | * |  | * |  | 5 | 360.19 | 122.25 | 0.00 | -174.93 |
| 93 |  | * |  | * |  | * | * | 6 | 361.39 | 123.45 | 0.00 | -174.45 |
| 94 |  | * |  |  | * | * |  | 6 | 364.46 | 126.52 | 0.00 | -175.99 |
| 95 |  | * |  |  | * | * | * | 7 | 365.36 | 127.42 | 0.00 | -175.36 |
| 96 |  |  | * | * |  | * |  | 5 | 366.68 | 128.74 | 0.00 | -178.17 |
| 97 |  |  | * |  |  | * | * | 5 | 367.68 | 129.74 | 0.00 | -178.67 |
| 98 |  |  | * | * |  |  | * | 5 | 368.27 | 130.33 | 0.00 | -178.96 |
| 99 |  |  |  | * | * | * | * | 7 | 368.31 | 130.37 | 0.00 | -176.83 |
| 100 |  | * |  | * | * |  |  | 6 | 368.90 | 130.96 | 0.00 | -178.21 |
| 101 |  |  | * |  |  | * |  | 4 | 370.36 | 132.42 | 0.00 | -181.07 |
| 102 |  | * |  | * | * |  | * | 7 | 370.64 | 132.70 | 0.00 | -178.00 |
| 103 |  |  |  | * | * | * |  | 6 | 370.68 | 132.74 | 0.00 | -179.10 |
| 104 |  |  | * | * |  |  |  | 4 | 372.16 | 134.22 | 0.00 | -181.97 |
| 105 |  | * |  |  |  | * |  | 4 | 372.62 | 134.68 | 0.00 | -182.20 |
| 106 |  | * |  |  |  | * | * | 5 | 373.43 | 135.49 | 0.00 | -181.54 |
| 107 |  | * |  | * |  |  |  | 4 | 376.60 | 138.66 | 0.00 | -184.19 |
| 108 |  |  |  | * |  | * | * | 5 | 376.95 | 139.01 | 0.00 | -183.30 |
| 109 |  |  |  |  | * | * |  | 5 | 377.07 | 139.13 | 0.00 | -183.36 |
| 110 |  | * |  | * |  |  | * | 5 | 378.16 | 140.22 | 0.00 | -183.91 |
| 111 |  |  |  |  | * | * | * | 6 | 379.21 | 141.27 | 0.00 | -183.36 |
| 112 |  | * |  |  | * |  | * | 6 | 379.42 | 141.48 | 0.00 | -183.47 |
| 113 |  |  |  | * | * |  |  | 5 | 379.92 | 141.98 | 0.00 | -184.79 |
| 114 |  |  |  | * |  | * |  | 4 | 381.25 | 143.31 | 0.00 | -186.51 |
| 115 |  |  |  | * | * |  | * | 6 | 381.81 | 143.87 | 0.00 | -184.66 |
| 116 |  |  | * |  |  |  |  | 3 | 382.24 | 144.30 | 0.00 | -188.05 |
| 117 |  |  | * |  |  |  | * | 4 | 384.24 | 146.30 | 0.00 | -188.00 |
| 118 |  | * |  |  | * |  |  | 5 | 392.46 | 154.52 | 0.00 | -191.06 |
| 119 |  |  |  |  |  | * |  | 3 | 393.53 | 155.59 | 0.00 | -193.70 |
| 120 |  |  |  |  | * |  | * | 5 | 393.80 | 155.86 | 0.00 | -191.73 |
| 121 |  |  |  | * |  |  |  | 3 | 393.98 | 156.04 | 0.00 | -193.92 |
| 122 |  |  |  | * |  |  | * | 4 | 395.38 | 157.44 | 0.00 | -193.58 |
| 123 |  | * |  |  |  |  | * | 4 | 395.45 | 157.51 | 0.00 | -193.61 |
| 124 |  |  |  |  |  | * | * | 4 | 395.52 | 157.58 | 0.00 | -193.64 |
| 125 |  |  |  |  | * |  |  | 4 | 399.33 | 161.39 | 0.00 | -195.55 |
| 126 |  | * |  |  |  |  |  | 3 | 413.34 | 175.40 | 0.00 | -203.60 |
| 127 |  |  |  |  |  |  | * | 3 | 421.30 | 183.36 | 0.00 | -207.58 |
| 128 |  |  |  |  |  |  |  | 2 | 428.33 | 190.39 | 0.00 | -212.13 |

**Supplementary Figure S5.** Maps of predicted population density of wild pigs for habitat occurring across Europe (a), Asia (b), Africa (c), Australia (d), North America (e), and South America (f). Figure 3. For terrestrial environments, areas of white represent low density (1 individual / km^2^), orange moderate density (6 individuals / km^2^), and dark red high density (≥11 individuals / km^2^). Maps were created using Google Earth Engine [^1^](#_ENREF_1) and QGIS 2.14.3 [^2^](#_ENREF_2).


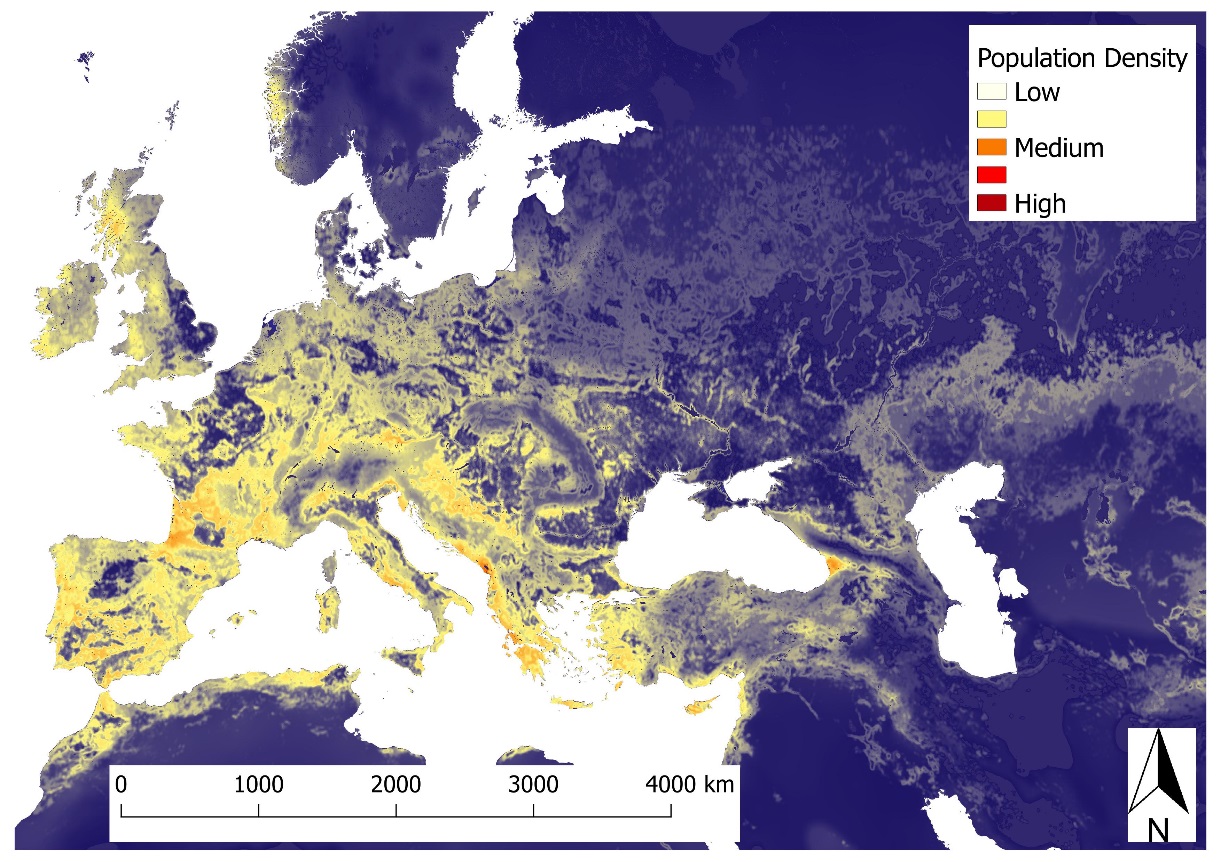


a. Europe


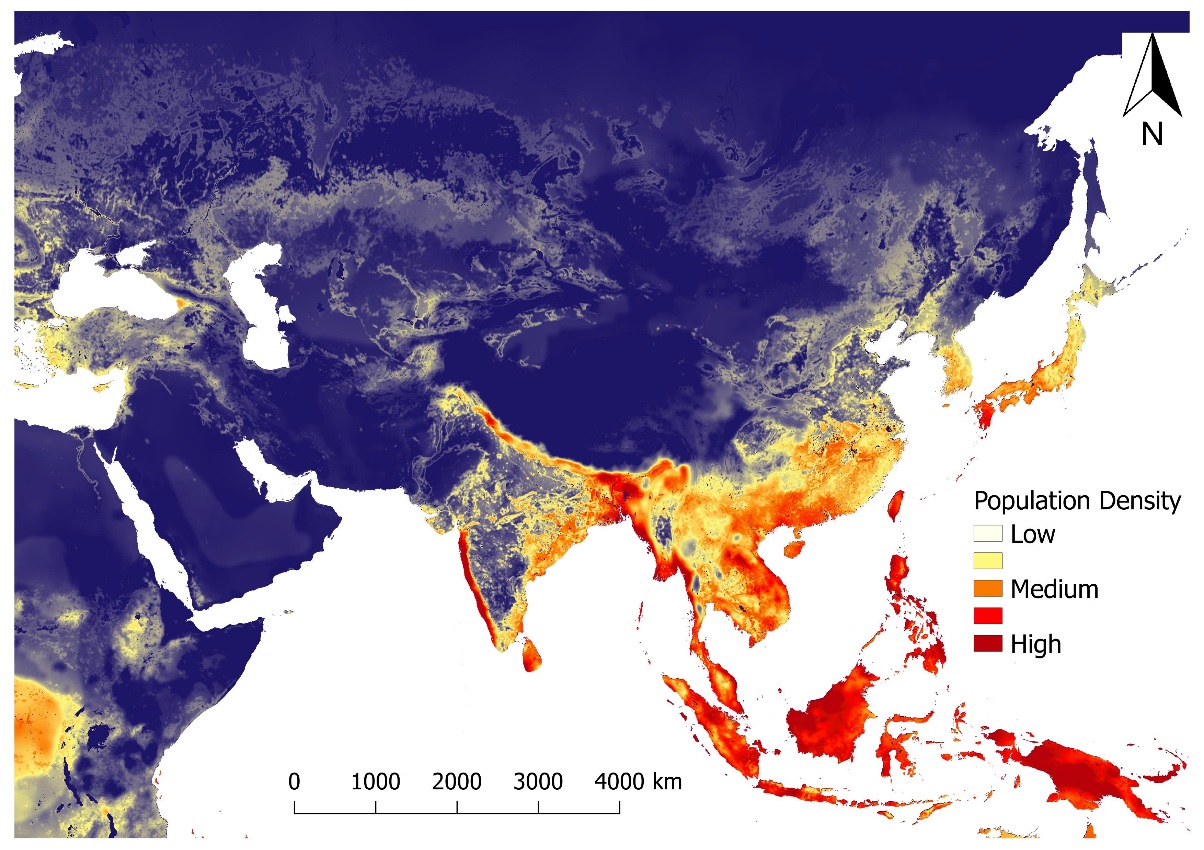


b. Asia


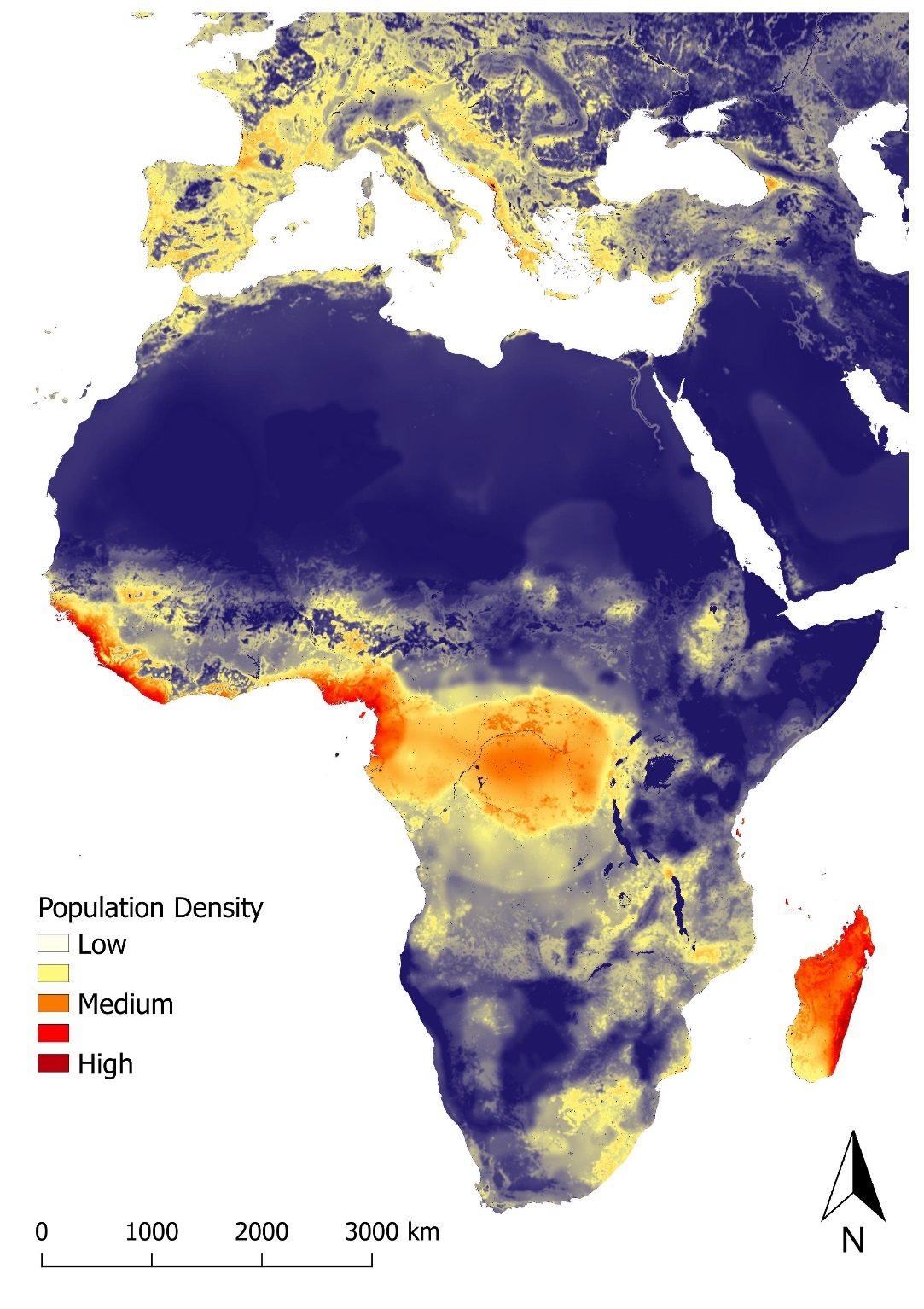


c. Africa


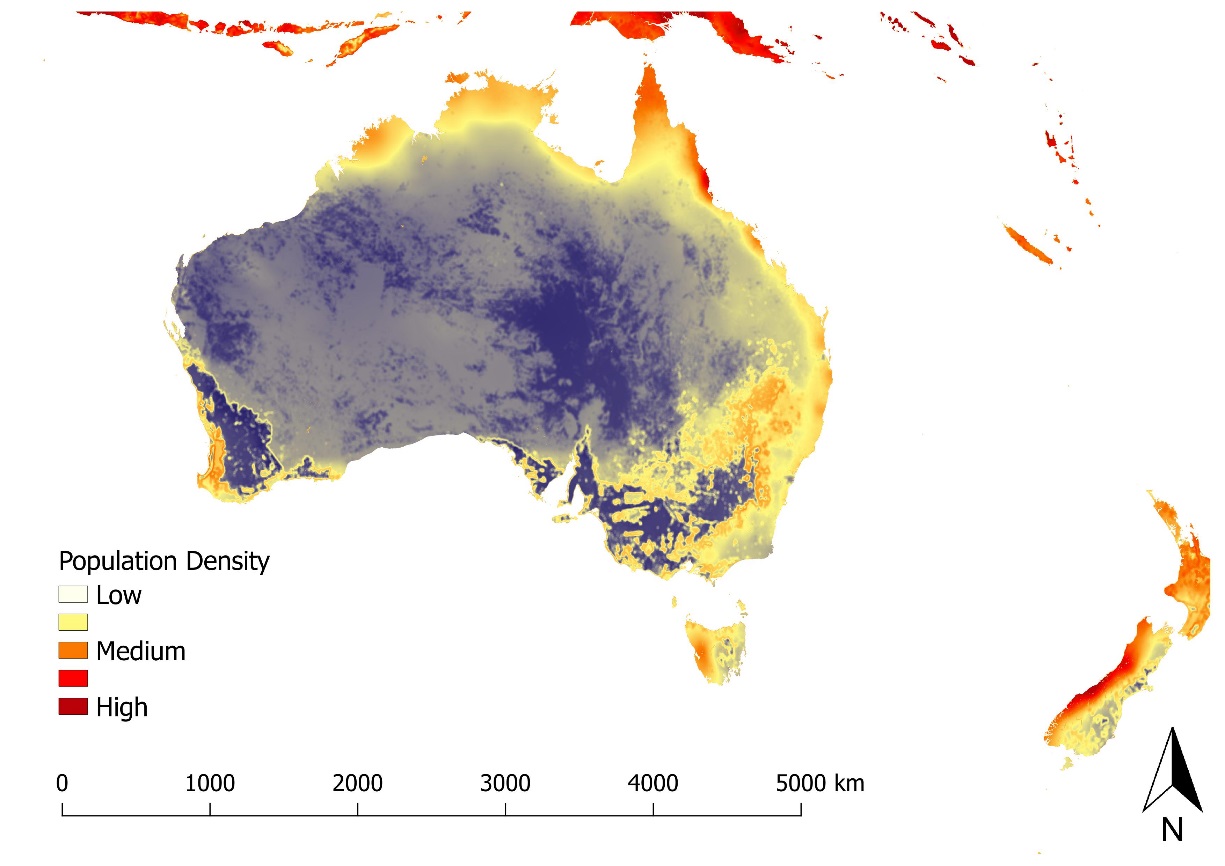


d. Australia


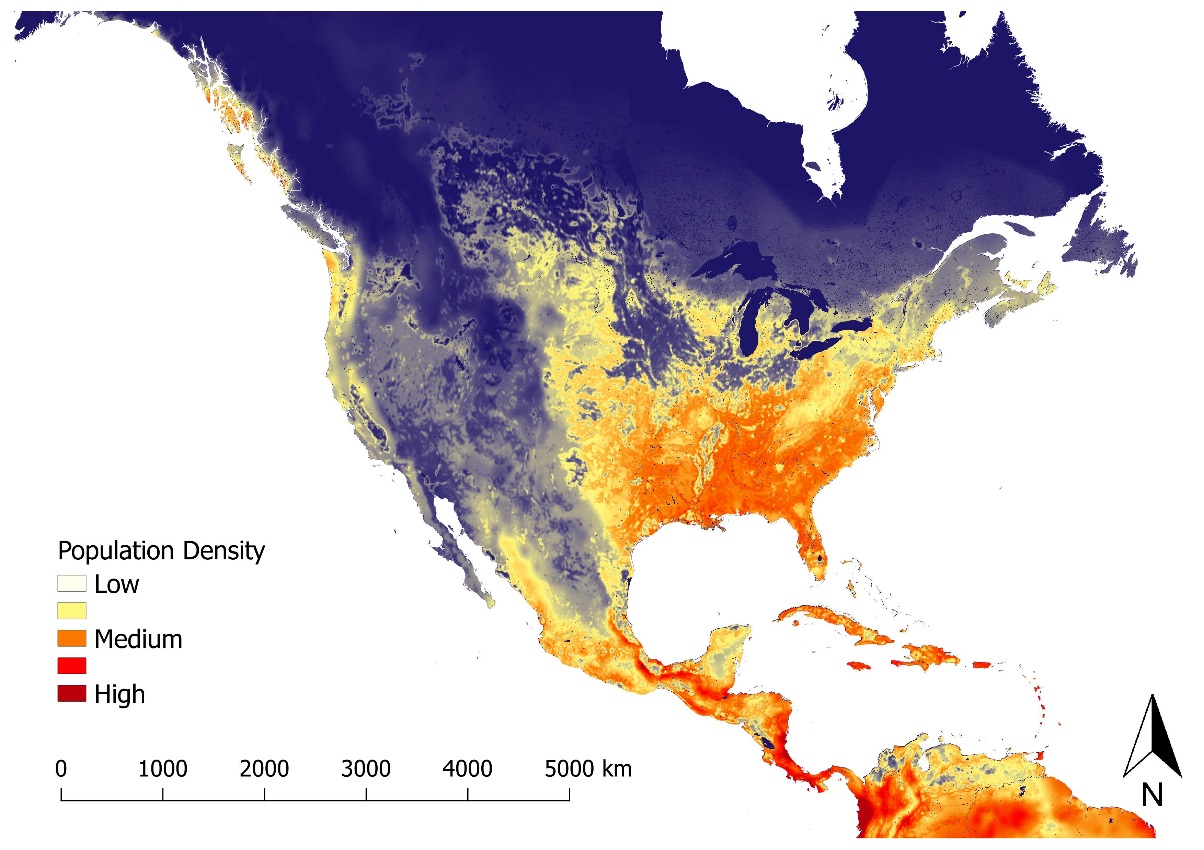


e. North America


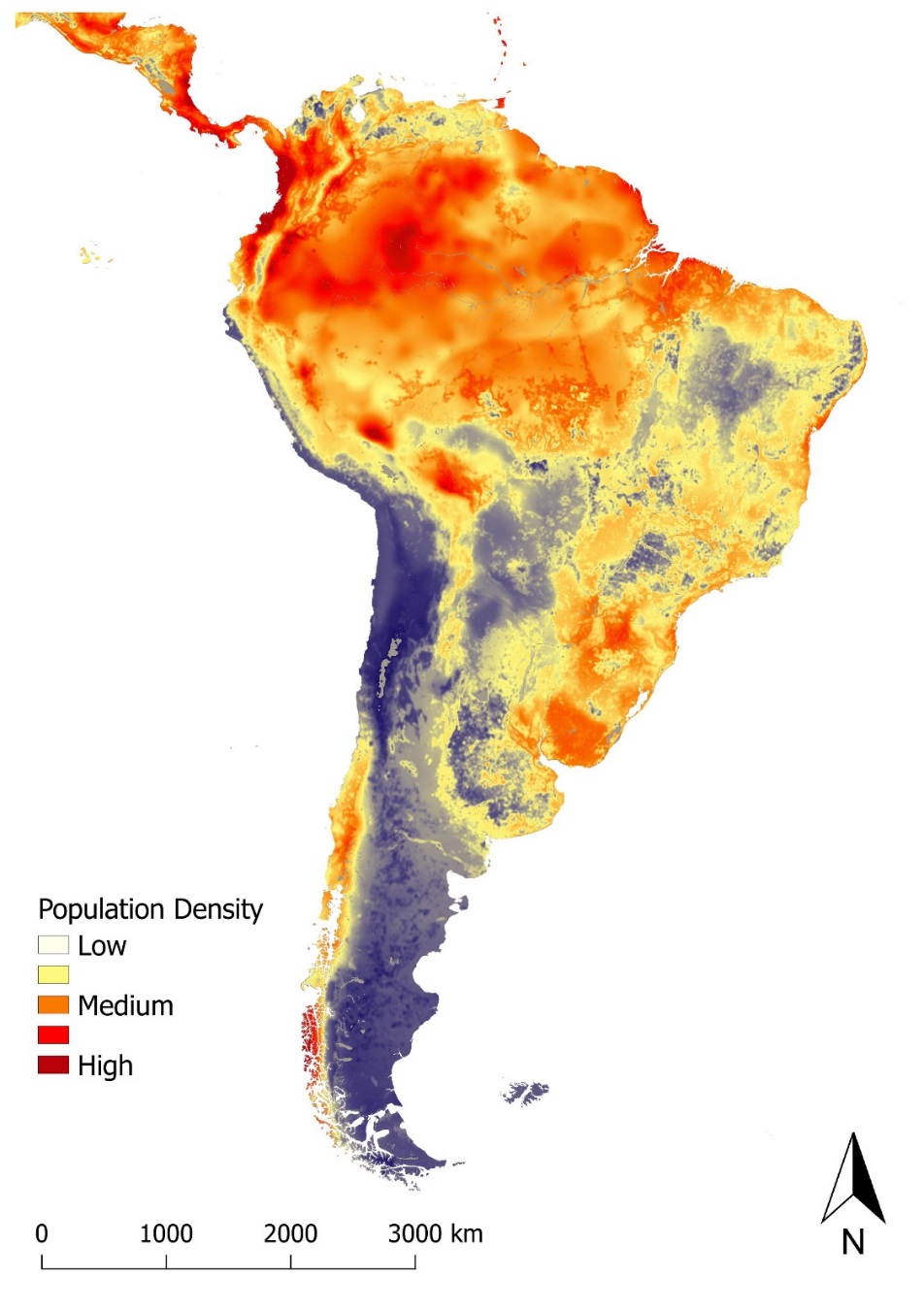


f. South America

**Literature Cited**
